# Salt-Induced Stabilization of EIN3/EIL1 Confers Salinity Tolerance by Deterring ROS Accumulation in *Arabidopsis*

**DOI:** 10.1371/journal.pgen.1004664

**Published:** 2014-10-16

**Authors:** Jinying Peng, Zhonghai Li, Xing Wen, Wenyang Li, Hui Shi, Longshu Yang, Huaiqiu Zhu, Hongwei Guo

**Affiliations:** 1 The State Key Laboratory of Protein and Plant Gene Research, College of Life Sciences, Peking University, Peking-Tsinghua Center of Life Sciences, Beijing, China; 2 Department of Biomedical Engineering, College of Engineering, and Center for Theoretical Biology, Peking University, Beijing, China; National University of Singapore and Temasek Life Sciences Laboratory, Singapore

## Abstract

Ethylene has been regarded as a stress hormone to regulate myriad stress responses. Salinity stress is one of the most serious abiotic stresses limiting plant growth and development. But how ethylene signaling is involved in plant response to salt stress is poorly understood. Here we showed that *Arabidopsis* plants pretreated with ethylene exhibited enhanced tolerance to salt stress. Gain- and loss-of-function studies demonstrated that EIN3 (ETHYLENE INSENSITIVE 3) and EIL1 (EIN3-LIKE 1), two ethylene-activated transcription factors, are necessary and sufficient for the enhanced salt tolerance. High salinity induced the accumulation of EIN3/EIL1 proteins by promoting the proteasomal degradation of two EIN3/EIL1-targeting F-box proteins, EBF1 and EBF2, in an EIN2-independent manner. Whole-genome transcriptome analysis identified a list of *SIED* (*Salt-Induced and EIN3/EIL1-Dependent*) genes that participate in salt stress responses, including several genes encoding reactive oxygen species (ROS) scavengers. We performed a genetic screen for *ein3 eil1*-like salt-hypersensitive mutants and identified 5 EIN3 direct target genes including a previously unknown gene, *SIED1* (At5g22270), which encodes a 93-amino acid polypeptide involved in ROS dismissal. We also found that activation of EIN3 increased peroxidase (POD) activity through the direct transcriptional regulation of *POD*s expression. Accordingly, ethylene pretreatment or EIN3 activation was able to preclude excess ROS accumulation and increased tolerance to salt stress. Taken together, our study provides new insights into the molecular action of ethylene signaling to enhance plant salt tolerance, and elucidates the transcriptional network of EIN3 in salt stress response.

## Author Summary

High salinity, as a world-wide abiotic stress, restricts root water uptake, damages cell physiology, and limits the productivity of agricultural crops. Ethylene is a major phytohormone that regulates plant development in response to adverse environments, including high salt stress. However, the molecular mechanisms of how ethylene signal exerts its effect and how ethylene signaling is modulated upon salt stress remain to be explored. Here, we report that high salinity induces EIN3/EIL1 protein accumulation and EBF1/2 protein degradation in an EIN2-independent manner. Moreover, the activated EIN3 deters excess ROS accumulation and increases salt tolerance. Transcriptome analysis and functional studies reveal an EIN3-directed gene network in salt stress response. Functional studies of 114 *SIED* (*Salt-Induced and EIN3/EIL1-Dependent*) genes identify a novel regulator of ROS dismissal and salt tolerance. This new understanding of ethylene/salt mutual regulation would allow a better manipulation and engineering of EIN3 and its downstream *SIED* genes to enhance plant tolerance and adaption to salt stress, particularly in those economically important crops in the future.

## Introduction

Soil salinity is a major abiotic stress that reduces plant growth and limits the productivity of agricultural crops. The detrimental effects of salt on plants are a consequence of both a water deficit resulting in osmotic stress and the effects of excess sodium ions imposed on critical biochemical processes [1]. The sessile nature of plants has favored the evolution of mechanisms to cope with various environmental stresses. One of these mechanisms is the release and utilization of a multitude of phytohormones, including a gaseous molecule ethylene [2].

Ethylene can trigger multiple physiological and morphological responses, including inhibition of cell expansion, induction of fruit ripening and abscission, and adaptation to stress conditions [3]. One of the well documented ethylene responses is the so-called “triple response” of etiolated seedlings, i.e. short, thickened root and hypocotyl, as well as exaggerated curvature of the apical hook [4]. Based on this highly reproducible and specific phenotype, a largely linear ethylene signal transduction pathway has been established [5]. In *Arabidopsis*, ethylene is perceived by a family of membrane-associated receptors [6], [7], [8], which are negative regulators of the signaling pathway, and ethylene binding leads to functional inactivation of the receptors [9]. In the absence of ethylene, the active receptors recruit CTR1 (CONSTITUTIVE TRIPLE RESPONSE1) to associate with the membrane and thus become activated [10], which subsequently represses the downstream signaling pathway mediated by ETHYLENE INSENSITIVE2 (EIN2) and EIN3. EIN2 is a central component of the ethylene signaling transduction pathway, and its null mutant *ein2* is completely insensitive to ethylene [11]. EIN2 is shown to locate in endoplasmic reticulum membrane [12], and undergoes a hormone-induced cleavage and translocation event that is controlled by CTR1-directed phosphorylation of its carboxyl-terminus [13], [14], [15]. As the requisite component for ethylene signaling, EIN2 positively regulates the functions of EIN3/EIL1 transcription factors, which results in the activation of transcription of *ERF1* and other downstream genes [16], [17]. EIN3/EIL1 are short-lived proteins, which are quickly stabilized and accumulate in the nucleus in the presence of ethylene. Genetic and biochemical studies revealed that EIN3/EIL1 are subject to ubiquitin/proteasome-mediated proteolysis that requires two F-box proteins, EBF1/EBF2 [16], [18], [19], [20]. Recently, our studies have demonstrated that ethylene stabilizes EIN3/EIL1 at least partly by promoting the proteasomal degradation of EBF1/EBF2, and that EIN2 is indispensable for mediating ethylene-induced EIN3/EIL1 accumulation and EBF1/2 degradation [18], highlighting the importance of EIN2 in the control of EIN3/EIL1 abundance.

In addition to its role in regulating plant growth and development, ethylene also plays a key role in plant responses to biotic and abiotic stresses [21]. Recently, the functions of components of ethylene signaling in salt stress response were investigated. The *ctr1-1* mutant exhibited increased salt tolerance and the germination rate and post-germination development of *ctr1-1* were more tolerant under salt and osmotic stress treatments, especially under high concentration of salt [22]. Under salt stress, the *ein2-1* mutant was severely affected in both seedling growth and seed germination processes, suggesting that EIN2 is required for salt stress tolerance [23], [24]. The *ein3-1eil1-1* double mutant exhibited remarkably reduced tolerance to high concentration of salt [22], [23], [24]. Recently, Jiang et al. reported that salinity-induced ethylene promotes Arabidopsis soil-salinity tolerance by enhancing Na/K homeostasis [25].

Despite such clear demonstration of a vital role of ethylene in salt stress response, the molecular mechanisms of how the ethylene signaling is modulated under salt stress condition and how ethylene signaling increases salinity tolerance are poorly understood. In this study, we demonstrated that plants pretreated with ethylene exhibited increased tolerance to salt stress, and that EIN3/EIL1 are both necessary and sufficient for salt tolerance. Interestingly, we found that salt stabilized EIN3/EIL1 protein by promoting EBF1/EBF2 proteasomal degradation in an EIN2 independent manner. Microarray analysis identified a large number of EIN3/EIL1-regulated genes (*SIED*s) that participate in salt stress response, including many genes encoding reactive oxygen species (ROS) scavengers. A novel EIN3 target gene, *SIED1*, was functionally studied and defined as an important mediator of ethylene-evoked salt tolerance.

## Results

### ACC/Ethylene Pretreatment or Activated Ethylene Signaling Increases Salt Tolerance

**Figure 1:**
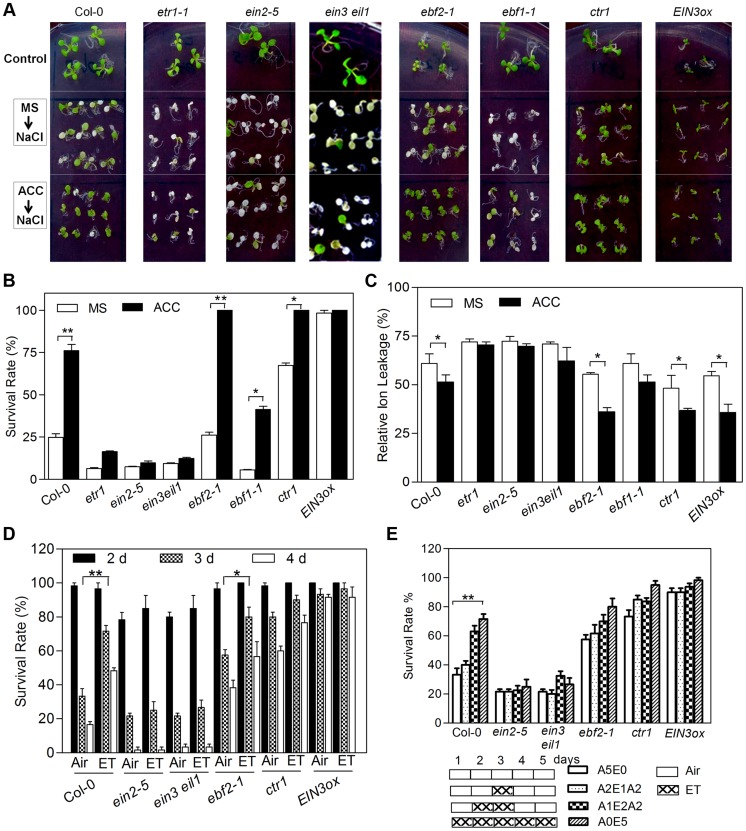
ACC/Ethylene pretreatment or enhanced ethylene signaling increases salt tolerance. (A) Plants were grown on MS medium with or without 10 µM ACC for 5 d and then transferred onto MS medium supplemented with 200 mM NaCl for 3 d. Plants were also transferred onto MS medium as controls. (B) Survival rate of plants shown in (A). Seedling death was scored as complete bleaching of cotyledons and leaves. Values are mean ± SD from 25 seedlings per replicate (*n* = 3 replicates). (Student's *t* test, *P<0.05 and **P<0.01). (C) Relative electrolyte leakage of plants shown in (A). Values are mean ± SD from 50 seedlings per replicate (*n* = 3 replicates). (Student's *t* test, *P<0.05 and **P<0.01). (D) Survival rate of plants pretreated with air (Air) or 20 ppm ethylene (ET) for 5 d and then transferred onto MS medium supplemented with 200 mM NaCl. Survival rates were calculated on the second, third and fourth day. Values are mean ± SD from 20 seedlings per replicate (*n* = 4 replicates). (Student's *t* test, *P<0.05 and **P<0.01). (E) Survival rate of plants pretreated with air or 20 ppm ethylene for indicated time and then transferred onto medium supplemented with 200 mM NaCl. Survival rates were calculated on the third day after transfer. Values are mean ± SD from 20 seedlings per replicate (*n* = 4 replicates). A5E0: 5 d of air treatment. A2E1A2: 2 d of air followed by 1 d of ethylene then 2 d of air treatment. A1E2A2: 1 d of air followed by 2 d of ethylene then 2 d of air treatment. A0E5: 5 d of ethylene treatment. ET: ethylene. (Student's *t* test, *P<0.05 and **P<0.01).

Previous studies investigating the effect of ethylene in salt stress were conducted in conditions where ethylene and salt stress were simultaneously applied [23]. Because several salt-induced seedlings responses, such as leaf epinasty, chlorophyll loss and growth retardation, are also regulated by ethylene [4], [5], it is sometimes not clear how the final morphological output is the result of an altered salt or ethylene response. To specifically ascertain the role of ethylene in salt response, we pretreated *Arabidopsis* seedlings with ethylene or its biosynthesis precursor ACC and then transferred to MS medium supplemented with 200 mM NaCl alone. Upon ACC pretreatment, wild-type Col-0 displayed enhanced tolerance to salt compared with untreated control, with higher survival rate and lower relative electrolyte leakage (an indicator for the salt stress damage) [26] (Figure 1A–C). By comparison, *ebf1-1* mutant showed slightly lower survival rate, whereas *ebf2-1*, an ethylene hypersensitive mutant [16], was more tolerant to salt than Col-0 upon ACC pretreatment (Figure 1A–C). Consistent with their respective ethylene response phenotype, *ctr1-1*, *EIN3ox* (a transgenic plant overexpressing EIN3) as well as *EIL1ox* displayed constitutively enhanced salt tolerance, whereas ACC pretreatment had virtually no effect on the salt tolerance of ethylene insensitive mutants, *etr1-1*, *ein2-5* and *ein3-1eil1-1* (Figure 1A–C; Figure S1). To further examine the effect of ACC pretreatment on salt tolerance, 5-day-old seedlings of wild-type, *ein3-1eil1-1* and *EIN3ox* were also transferred onto MS medium supplemented with serial concentrations of NaCl (0, 50, 100, 150 and 200 mM). Similarly, ACC pretreatment significantly increased the survival rate, fresh weight as well as root length of wild-type but not *ein3-1eil1-1* compared with ACC-untreated plants when 100 mM or higher concentrations of NaCl were applied (Figure S2E *vs* S2B, S2F *vs* S2C and S2G *vs* S2D). Consistently, *EIN3ox* seedlings showed constitutively increased salt tolerance in terms of survival rate, fresh weight and root length (Figure S2E *vs* S2B, S2F *vs* S2C and S2G *vs* S2D). Together, these results demonstrate that ACC pretreatment or overexpression of *EIN3* leads to increased tolerance to salt stress, which depends on the canonical ethylene signaling pathway.

To exclude the possibility that the observed effect of ACC pretreatment was due to the residual ACC remained in the pretreated seedlings, the experiment was repeated using ethylene gas, which was quickly diffusing away. After 5 days of 10 ppm ethylene gas treatment, seedlings were transferred onto MS medium supplemented with 200 mM NaCl in the air, and survival rates were calculated after two, three and four days, respectively. We found that ethylene pretreatment effectively increased the tolerance to salt in wild-type, *ebf2-1* and *ctr1-1*, evidenced by higher survival rates after three or four days of salt treatment, but had little effect on *ein2-5* and *ein3-1eil1-1* (Figure 1D). Ethylene pretreatments with different lengths of time were also investigated. Seedlings pretreated with ethylene for 2 or 5 days exhibited increasingly enhanced survival rate in Col-0, *ebf2-1*, but not in *ein2-5* and *ein3-1eil1-1*, while 1 day of ethylene pretreatment had only marginal effect (Figure 1E). Together with the ACC pretreatment experiments, these results support that exogenous ethylene application beforehand effectively increases salt tolerance.

To further study the function of EIN3/EIL1 in ethylene-mediated salt response, a transgenic line expressing estradiol-inducible EIN3-FLAG in the *ein3 eil1 ebf1 ebf2* quadruple mutant (*iE*/*qm*) was investigated [18]. Previous studies demonstrated that the *iE/qm* seedlings were completely insensitive to exogenously applied ethylene, and the accumulation of EIN3-FLAG fusion protein can be induced by estradiol (but not by ethylene) in a dose-dependent manner [18]. As reported, the EIN3-FLAG protein was undetected in estradiol-untreated *iE/qm*, and it was evidently induced in *iE/qm* upon estradiol treatment, but ACC treatment did not further increase its protein accumulation (Figure S3A, Lane 1, 2, 8, or Lane 7, 9). We also found that salt treatment did not affect EIN3 protein level in estradiol-treated *iE/qm* regardless of treatment time (Figure S3A, Lane 3–5, or Lane 2, 6). These results demonstrated that the estradiol-induced EIN3 protein accumulation in *iE/qm* seedlings is not altered by ethylene or salt treatment.

Next, we investigated whether the estradiol-induced EIN3 protein in *iE/qm* seedlings effectively increased the tolerance to salt stress. In the absence of estadiol, where EIN3 protein was not detectable, the cotyledons and leaves of *iE/qm* seedlings treated with 200 mM NaCl were severely bleached after 3 days (Figure S3B), and the survival rate was declined to less than 30% (Figure S3C). In contrast, in the presence of serial concentrations of estradiol (from 0.1 to 20 µM), *iE/qm* seedlings treated with 200 mM NaCl for 3 days appeared largely green and healthy (Figure S3B), and the survival rates remained over 90% in all cases (Figure S3C). Despite all concentrations of estradiol were sufficient for conferring salt tolerance, we noted that *iE/qm* seedlings supplemented with lower concentrations (0.1 or 1 µM) grew better on salt medium (Figure S3B). Therefore, all subsequent physiological experiments were performed with 1 µM estradiol. In line with cotyledon yellowing phenotype and survival rate, the leaf chlorophyll content and fresh shoot weight were significantly higher in estradiol-treated *iE/qm* seedlings than the untreated control under salt stress condition (Figure S3D, S3F). Conversely, upon salt treatment, the ion leakage was evidently lower in estradiol-treated *iE/qm* plants than the untreated control (Figure S3E). Taken together, these results indicate that loss of EIN3/EIL1 function leads to hypersensitivity to salt stress whereas accumulation of EIN3 alone results in enhanced salt tolerance, highlighting the requirement and sufficiency of EIN3/EIL1 for salt tolerance in *Arabidopsis*.

### High Salinity Enhances EIN3 Protein Accumulation and Transcriptional Activity in Both EIN2-Dependent and EIN2-Independent Manners

**Figure 2:**
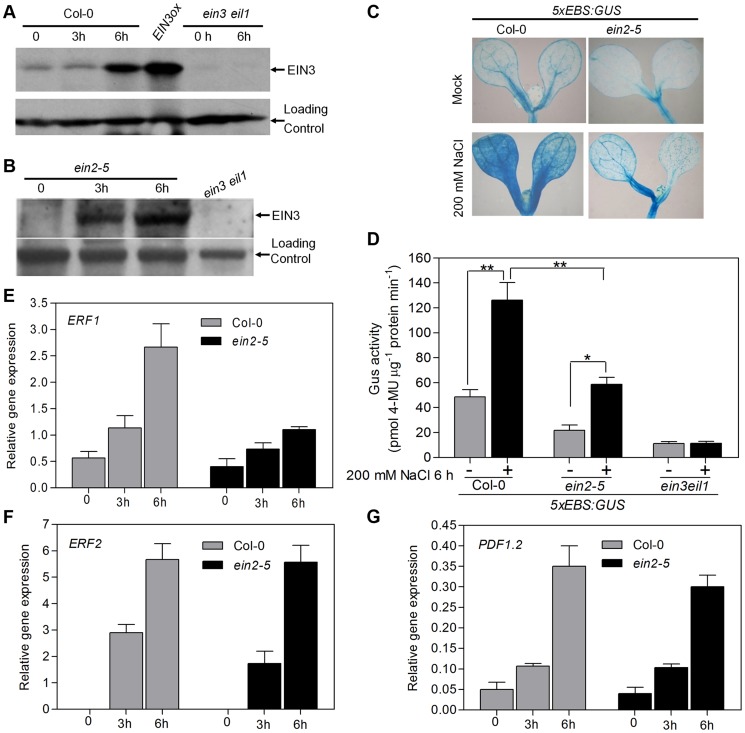
Salt treatment promotes protein accumulation and transcriptional activity of EIN3 in both EIN2-dependent and EIN2-independent manners. (A) Salt treatment promotes EIN3 protein accumulation in wild type. 5-d-old seedlings were treated with 200 mM NaCl for 3 h and 6 h. Protein was extracted and subjected to immunoblots using anti-EIN3 antibody. A nonspecific band was used as a loading control. (B) Salt treatment promotes EIN3 protein accumulation in *ein2-5* mutant. Experiments were repeated three times with similar results. (C) Histochemical analysis of *5xEBS:GUS* transgenic plants. (D) *5xEBS:GUS* activity in Col-0 or *ein2-5* background was measured. Two biological replicates and three technical replicates were performed (Student's *t* test, *P<0.05 and **P<0.01). (E–G) Real-time RT-PCR analysis of gene expression of *ERF1* (E), *ERF2* (F) and *PDF1.2* (G).

Given that EIN3 is a critical regulator of plant salt responses, we next determined whether, and if so, how EIN3 is modulated by salt stress. We first monitored the level of endogenous EIN3 protein using an anti-EIN3 antibody [16], [18] in response to salt treatment. We found that the levels of EIN3 protein started to increase after 3 h of salt treatment and dramatically accumulated after 6 h of treatment in wild-type Col-0 (Figure 2A). We also checked the levels of *EIN3* mRNA and found no obvious change after 6 h of salt treatment (Figure S4), suggesting that salt regulates EIN3 accumulation at the protein level.

Previous study indicated that EIN2 is absolutely required for ethylene-induced EIN3/EIL1 accumulation, as no EIN3 or EIL1 protein can be detected in *ein2* mutant [16], [18]. To determine whether EIN2 is required for salt-induced EIN3/EIL1 protein accumulation, we detected the EIN3/EIL1 protein level in *ein2-5* mutant under salt treatment. Surprisingly, we found that salt treatment (but not mock treatment, Figure S5A) promoted EIN3 and EIL1 proteins accumulation in the *ein2-5* mutant background, although not as dramatic as in wild-type (Figure 2B and Figure S6). These results indicate that the salt stress signal is able to promote EIN3/EIL1 proteins accumulation in an EIN2-independent manner. In addition, we also analyzed the EIN3 protein levels in an ethylene receptor mutant *etr1-1* [9] upon treatment with 200 mM NaCl for 3 h and 6 h using anti-EIN3 antibody. We found that EIN3 protein was evidently induced in *etr1-1* mutants upon salt treatment for 6 h (Figure S5B), suggesting that salt induced EIN3 protein accumulation does not require the canonical ethylene perception.

We next investigated whether salt-induced EIN3 protein is transcriptionally functioning. A transgenic reporter line that harbors the GUS report gene driven by five tandem repeats of the EIN3 binding site (EBS) followed by the minimal 35S promoter, *5xEBS:GUS*, has been previously used to monitor the transcriptional activity of EIN3 [27], [28]. Upon salt treatment, GUS staining became overly intensified in the cotyledons and hypocotyls of *5xEBS:GUS*/Col-0 plants (Figure 2C), indicative of elevated levels of EIN3 activity under this condition, which was further supported by a quantification assay (Figure 2D). Compared to that in Col-0 background, GUS activity was also evidently up-regulated in *5xEBS:GUS*/*ein2-5* plants upon salt treatment, although to a lesser extent (Figure 2C and 2D). By contrast, GUS activity in *5xEBS:GUS*/*ein3 eil1* plants did not increase upon salt stress (Figure 2D), suggesting that salt-increased 5xEBS:GUS activity is EIN3/EIL1-dependent. We also observed that the expression levels of several ethylene responsive genes, including *ERF1*, *ERF2* and *PDF1.2*, were up-regulated by salt in both wild type and *ein2-5* mutants (Figure 2E–G). In keeping with the results of EIN3 accumulation and GUS expression (Figure 2B–D), the expression level of *ERF1*, a direct target gene of EIN3 [17], was also lower in salt-treated *ein2-5* mutant compared with that in wild-type (Figure 2E). Thus, although salt treatment did promote EIN3 protein accumulation in *ein2-5*, the relative lower level and activity of EIN3 might be inadequate to compensate for the loss of EIN2 that could elicit additional pathways contributing to salt tolerance. Taken together, our results suggest that, in addition to the canonical EIN2-dependent pathway, there exists a new pathway independent of EIN2 to mediate the salt stress signal to promote EIN3/EIL1 protein accumulation.

### Salt Promotes EBF1/EBF2 Protein Degradation in an EIN2-Independent Manner

**Figure 3:**
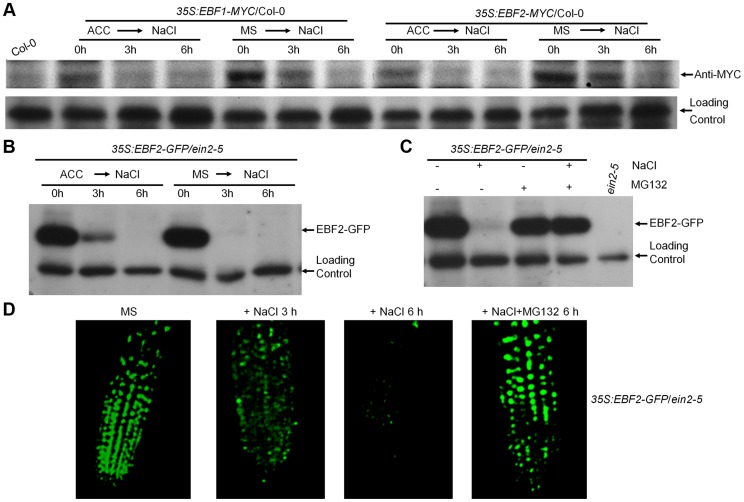
Salt treatment promotes EBF1/EBF2 protein degradation in an EIN2-independent manner. (A) Immunoblot assays of EBF1/2-MYC protein in Col-0. Transgenic seedlings overexpressing EBF1/2-MYC in Col-0 grown on MS medium supplemented with or without 10 µM ACC for 5 d were subjected to 200 mM NaCl for 3 h and 6 h. A nonspecific band was used as a loading control. (B) Immunoblot assay of EBF2-GFP protein in *ein2-5* background. Transgenic seedlings overexpressing EBF2-GFP in *ein2-5* background grown on medium supplemented with or without 10 µM ACC for 5 d were subjected to 200 mM NaCl for 3 h and 6 h. Experiments were repeated three times with similar results. (C) Salt induced EBF2 protein degradation was inhibited by MG132. 5-d-old plants were treated with 200 mM NaCl or/and 50 µM MG132 for 6 h. Experiments were repeated three times with similar results. (D) GFP fluorescence of *35S:EBF2-GFP* in the roots of *ein2-5* mutant. The seedlings grown on MS medium for 5 d were treated with 200 mM NaCl and/or 50 µM MG132 for 6 h.

It has been established that the stability of EIN3 is controlled by two F-box proteins, EBF1/EBF2, and that ethylene-induced EIN3 stabilization is at least partly mediated by the destabilization of EBF1/EBF2 proteins in an EIN2-dependent manner [16], [18], [19], [20]. To further characterize how EIN3 accumulation is enhanced by salt, we examined the levels of EBF1/EBF2 protein after salt treatment. Our initial effort to produce polyclonal antibodies recognizing endogenous EBF1 or EBF2 protein in plant tissues was unsuccessful. Therefore, two transgenic lines, *35S:EBF1-MYC*/Col-0 and *35S:EBF2-MYC*/Col-0 [18], were used to detect the EBF1 and EBF2 protein levels. Immunoblot analysis showed that the protein levels of EBF1-MYC and EBF2-MYC markedly decreased upon salt treatment (Figure 3A). We also noted that ACC pretreatment seemed to reinforce the destruction of EBF1/EBF2 proteins, as seedlings pretreated with ACC accumulated less EBF1/EBF2 proteins after salt application (Figure 3A). Together with the finding that EIN3 protein level is not altered by salt in *iE/qm* seedlings (Figure S3A), these results suggest that the salt-induced accumulation of EIN3 protein is due to reduced levels of EIN3-targeting F-box proteins.

Our above data showed that salt induced EIN3 protein accumulation in *ein2-5* mutant, so we asked whether salt-induced destruction of EBF1/EBF2 proteins also take place in the absence of EIN2 function. To address this question, a previously generated transgenic line, *35S:EBF2-GFP/ein2-5*, which showed high level of EBF2-GFP accumulation [18], was used. Immunoblot analysis showed that the protein levels of EBF2-GFP markedly decreased upon salt treatment, regardless of ACC pretreatment (Figure 3B). By contrast, treatment with MG132, a 26S proteasome inhibitor, promoted a dramatic accumulation of EBF2-GFP and reversed the salt-induced EBF2-GFP degradation (Figure 3C). Similarly, GFP fluorescence was dramatically reduced in the *35S:EBF2-GFP*/*ein2-5* after salt treatment, but MG132 treatment effectively reversed the salt effect and stabilized EBF2-GFP protein (Figure 3D). We further examined the effect of other salt ions on EBF2 stability, and found that, as NaCl, treatments of KCl, NaNO_3_ and KNO_3_ all similarly led to the destruction of EBF2-GFP protein in *ein2-5* mutant background (Figure S7A). We also excluded the involvement of osmotic stress in the control of salt-induced EBF protein degradation, as high dose of mannitol treatment (200 mM) had no effect on EBF2-GFP stability (Figure S7B). Collectively, our data clearly demonstrated that salt stress leads to the proteasome-mediated degradation of EBF1/EBF2 proteins independent of the upstream ethylene signaling components, such as EIN2.

### Transcriptome Profiling Analyses Identify Salt-Regulated EIN3/EIL1-Dependent Genes

Our above data indicated that EIN3/EIL1 are both necessary and sufficient for conferring enhanced salt tolerance. To elucidate the molecular network underlying EIN3/EIL1-induced salt tolerance, we performed transcriptome profiling of *EIN3ox*, *ein3eil1* and wild type Col-0. For this analysis, 5-day-old light-grown seedlings treated with or without 200 mM NaCl for 6 h were used. This design enabled us to compare the transcriptional profiles among plants with different levels of EIN3 activity, as well as to identify salt-regulated and EIN3/EIL1-dependent genes.

**Figure 4:**
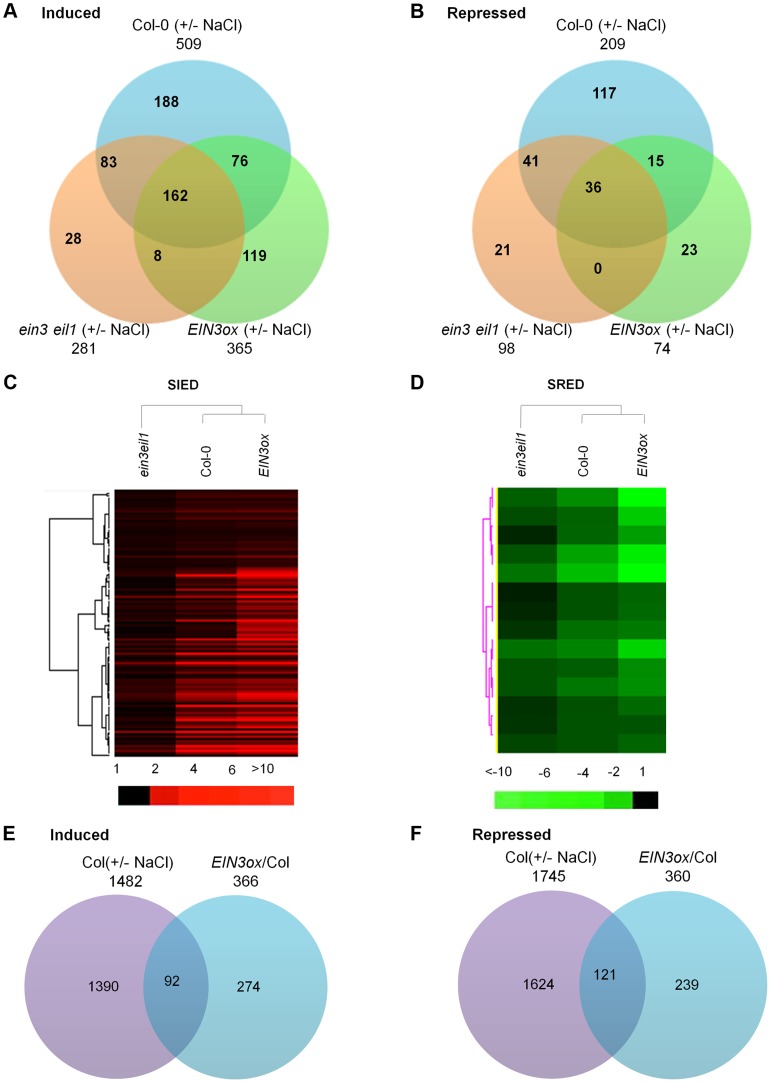
Transcriptome profiling analyses identify salt-regulated EIN3/EIL1-dependent genes. (A) and (B) Venn diagrams showing the overlaps among transcripts induced or repressed (q<0.05 and 5-fold change as a cutoff) by salt in Col-0, *ein3eil1* and *EIN3ox* plants. (C) and (D) Hierarchical clusters displaying the salt-induced expression of those SIED (Salt-Induced EIN3/EIL1-Dependent) and SRED (Salt-Repressed EIN3/EIL1-Dependent) genes in Col-0, *ein3eil1* and *EIN3ox* plants. A total of 114 SIED genes (C) and 14 SRED genes (D) were included in the cluster (Table S1 and S2). (E) and (F) Venn diagrams showing the overlaps between transcripts induced or repressed (2-fold cutoff) by salt in Col-0 and transcripts induced or repressed (2-fold cutoff) by overexpression of *EIN3* (comparing transcriptome of *EIN3ox* versus Col-0 under unstressed condition).

Treatment with high salt for 6 h resulted in the induction of 1482 transcripts while the repression of 1745 transcripts (using both q value<0.05 and 2-fold as a cutoff) in wild-type Col-0 (Figure 4E, 4F). Applying a q value<0.05 and 5-fold as a cutoff, 509 transcripts were induced while 209 transcripts were repressed in wild type by salt treatment, which were arbitrarily defined as salt-regulated genes in this study (Figure 4A and 4B). Using the same cutoff, 365 and 74 transcripts in *EIN3ox* while 281 and 98 transcripts in *ein3eil1* were induced and repressed by salt treatment, respectively (Figure 4A, 4B). Of the 509 salt-induced genes, 162 were also elevated in salt-treated *EIN3ox* and *ein3eil1* mutant (Figure 4A). Conversely, 36 out of 209 salt-repressed genes were also down-regulated in salt-treated *EIN3ox* and *ein3eil1* mutant (Figure 4B). To investigate how EIN3 activation leads to increased salt tolerance, we were particularly interested in two classes of genes: salt-induced EIN3/EIL1-dependent (SIED) genes and salt-repressed EIN3/EIL1-dependent (SRED) genes (Figure 4C, 4D). The former class includes those genes whose levels are induced by salt at least 5-fold in Col-0 (P value 0.0041), plus that salt induction is more pronounced in *EIN3ox* (P value 0.00099) but less in *ein3 eil1* (P value 0.0094) (i.e. salt-induced gene expression is at least partly dependent on EIN3 activity) (Table S1). The latter class includes those genes whose levels are repressed by salt at least 5-fold in Col-0 (P value 0.0016), plus that salt repression is more pronounced in *EIN3ox* (P value 0.00093) but less in *ein3 eil1* (P value 0.0081) (Table S2).

Based on these criteria, 114 SIED genes and 14 SRED genes were identified (Table S1 and S2). The drastic difference on the number of SIED and SRED genes suggested that EIN3/EIL1 might enhance salt tolerance mainly through inducing genes or pathways that participate in plant survival, rather than repressing genes or pathways that lead to plant death under salinity stress. In support of this speculation, 18 out of 114 SIED genes (∼16%) are defense-related genes that function to enhance plant tolerance or resistance to abiotic or biotic stresses. Several ERF (11) and JAZ (3) genes were found to be SIED genes, implying that the signaling pathways of ethylene and jasmonic acid (JA), two stress hormones, have been preferentially activated by salt stress, which is consistent with previous studies [23], [29], [30]. The considerable enrichment of *ERF* genes, many of which are direct target genes of EIN3 [17], [31], suggests that the identification of *SIED* genes is biologically relevant. Furthermore, when compared the *SIED*s with ethylene-regulated EIN3-target genes identified by ChIP-Sequencing [32], we found that 15 out of 114 *SIED*s (Highlighted in Table S1), such as *At5g22270* and *At5g59820* (*ZAT12*), are the direct targets of EIN3. However, most of *SIED*s are not the target genes of EIN3 identified by Chang et al. [32]. This could be due to that EIN3 preferentially binds to specific subsets of target promoters dependent on the initial treatment/stimulus. Alternatively, it is also possible that the majority of *SIED* genes are indirectly induced by EIN3, for instance, via the ERF transcription factors. The *SIED* genes were further analyzed using the gene ontology (GO) enrichment tool Gorilla [33]. We found that, in terms of molecular function category, there were notable enrichments for metabolic processes, as well as transcription, DNA binding, and oxidoreductase activity (Figure S8). For instance, of 114 SIED genes, we found 9 genes encoding oxidoreductases and 4 genes involved in electron transport or energy pathways, suggesting that modulation of oxidative/reductive status under salt stress might be an important mechanism of EIN3/EIL1 action to enhance plant survival.

In this study, we demonstrated that pretreatment with ethylene conferred increased salt tolerance, which depends on the action of EIN3/EIL1 (Figure 1). One explanation for this priming effect of ethylene is that EIN3/EIL1 activation in advance alters the expression of genes that ultimately leads to salt tolerance. To test this possibility, we compared the salt-regulated transcriptome (salt-treated versus untreated Col-0) and EIN3-regulated transcriptome (*EIN3ox* versus Col-0 without salt treatment). By a 2-fold cutoff, 366 genes (P value 0.0096) were identified as EIN3-induced while 360 genes (P value 0.0099) were EIN3-repressed based on transcriptome profiling (Figure 4E, 4F). We found that 92 out of 366 EIN3-induced genes (∼25%) and 121 out of 360 EIN3-repressed genes (∼34%) were also induced and repressed by salt stress, respectively (Figure 4E, 4F). By a 5-fold cutoff, 17 out of 62 genes (∼27%) vastly up-regulated by EIN3 (*EIN3ox* versus Col-0) (P value 0.0095), were also highly induced by salt, including several *ERFs*, defense genes, and biosynthetic process and metabolism genes (Table S3). Six genes were selected to further verify the microarray data using qRT-PCR, which showed largely similar expression patterns (Figure S9). These results indicated that overexpression of *EIN3* activated the expression of a number of stress-responsive defense and metabolism genes even under unstressed conditions. Therefore, the priming effect of ACC/ethylene pretreatment could be attributed to altered expression of numerous salt-responsive genes, which subsequently increases tolerance when salt stress is encountered. Further investigation on the functionality of these stress-responsive defense and metabolism genes in salt tolerance is needed to test this hypothesis.

### Functional Studies of *SIED* Genes Identify a Novel Regulator of Salt Tolerance

**Figure 5:**
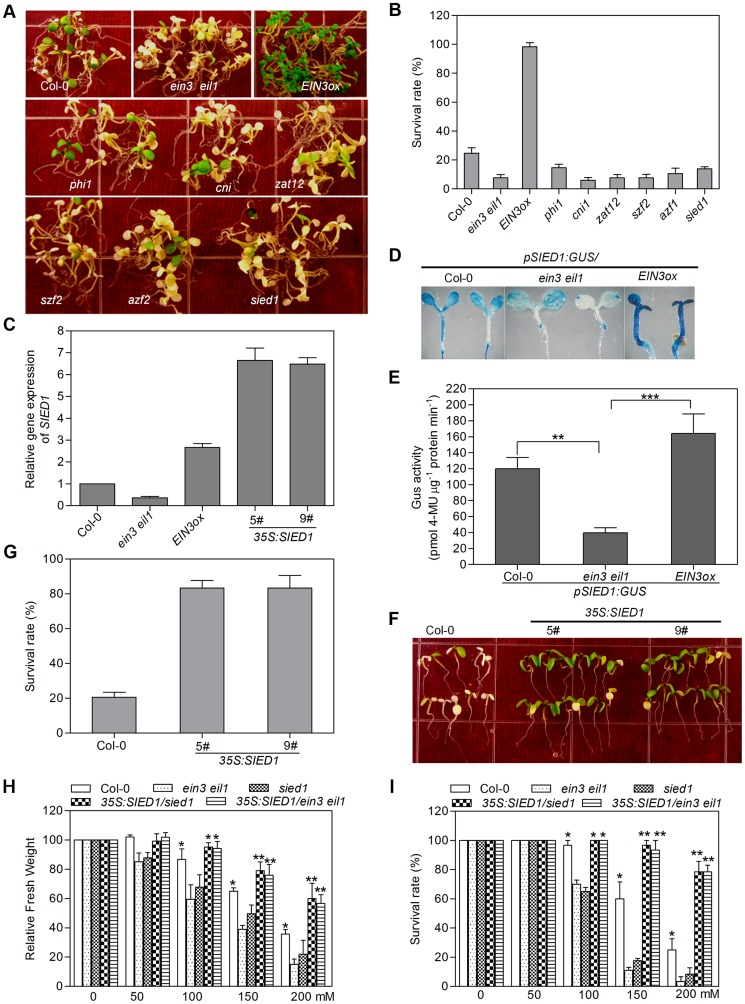
Functional characterization of *SIED* genes identifies a novel regulator of salt tolerance. (A) Plants were grown on MS medium for 5 d and then transferred onto MS medium supplemented with 200 mM NaCl for 4 d. Experiments were repeated three times with similar results. (B) Survival rate of plants shown in (A). Values are mean ± SD from at least 50 seedlings per replicate (*n* = 4 replicates). (C) qRT-PCR analysis of *SIED1* expression. (D) Histochemical analysis of *SIED1* expression in Col-0, *ein3eil1* and *EIN3ox* plants. (E) *pSIED1:GUS* activity in Col-0, *ein3eil1* or *EIN3ox* background (Student's *t* test, **P<0.01 and ***P<0.001). (F) Overexpression of SIED1 in wild-type enhanced salt tolerance. Seedlings were grown on MS medium for 5 d and then transferred onto MS medium supplemented with 200 mM NaCl for 4 d. Experiments were repeated three times with similar results. (G) Survival rate of plants shown in (F). Values are mean ± SD from at least 50 seedlings per replicate (n = 3 replicates). (H) and (I) Overexpression of SIED1 in ein3eil1 or sied1 backgrounds enhanced salt tolerance. Fresh weight (H) and survival rate (I) were measured (Student's t test, **P<0.01 and ***P<0.001).

To further investigate the roles of the *SIED* genes in salt tolerance, Salk T-DNA insertion lines of *SIED* genes were ordered from ABRC, and the homozygous lines of 47 insertion mutants were obtained and verified by genotyping (Table S4). Characterization of salt stress phenotype showed that, while 41 mutants were indistinguishable from wild type, 6 mutants, namely *zat12*, *azf2*, *cni1*, *szf2*, *phil* and SALK_067396 (hereafter designated as *sied1*, *salt-induced and EIN3/EIL1-dependent gene 1*), exhibited a salt-hypersensitivity phenotype similar to *ein3 eil1*, which showed low survival rate under salt stress (Figure 5A, 5B). PCR genotyping assays showed that the six mutant lines were knockout alleles in their corresponding genes (Figure S10), suggesting that these genes are positive regulators of salt tolerance.

Interestingly, five of these *SIEDs*, *ZAT12* [34], [35], [36], *AZF2* [37], *SZF2* [38], *CNI1* [39] and *PHI1* [40] have been previously demonstrated to modulate various abiotic stresses. For instance, transgenic plants overexpressing *ZAT12* were more tolerant to osmotic stress, while *zat12* knockout mutants were more sensitive to osmotic and salt stress [34]. Overexpression of *CNI1* (Carbon/Nitrogen Insensitive 1), a RING-type ubiquitin ligase, caused a hyposensitivity to C/N stress, and *cni1* knockout mutants resulted in hypersensitivity to C/N stress and salt treatment [39]. Of the five genes, three genes encode zinc-finger transcription factors (ZAT12, AZF2, SZF2). In fact, we have identified at least 9 genes encoding zinc-finger transcriptional regulators as *SIED* (Table S1). It thus remains interesting to determine whether all other identified zinc-finger proteins are involved in salt tolerance. Together, our results supported the idea that many EIN3/EIL1 target genes identified in this analysis are involved in various stress responses, including salt stress.

The sixth salt-hypersensitivity mutant, *sied1*, corresponds to At5g22270, a functionally unknown gene encoding a 93-amino acid polypeptide. Microarray data and qRT-PCR analysis showed that *SIED1* had evidently higher expression level in *EIN3ox* and lower level in *ein3 eil1* compared with that of wild type (Figure 5C). To confirm its EIN3-induced expression pattern, we generated a transgenic reporter line that harbors the β-*glucuronidase* (*GUS*) gene driven by the promoter of *SIED1*. Consistent with the gene expression data, GUS staining was weaker in *ein3 eil1* but stronger in *EIN3ox* than that in wild type (Figure 5D, 5E). Moreover, we found that EIN3 directly binds to the promoter of *SIED1*, as well as other *SIED* genes including *ZAT12*, *SZF2*, *PHI* and *AZF2*, but does not bind to the promoter region of *CNI1* (Figure S11E–F), indicating that EIN3 selectively binds to the promoters of many *SIED* genes *in vivo*.

Since loss-of-function *SIED1* mutation led to salt hypersensitivity, we next generated transgenic plants constitutively overexpressing *SIED1* in wild type to further investigate its role in salt tolerance. qRT-PCR analysis revealed that higher levels of *SIED1* mRNA were detected in two independent overexpression lines 5# and 9# compared with Col-0 (Figure 5C). Phenotypic analysis showed that overexpression of *SIED1* effectively enhanced salt tolerance and greatly increased survival rate upon salt treatment for 4 days (Figure 5F, 5G). Since *SIED1* is a direct target of EIN3, we then determined whether overexpression of *SIED1* could repress the salt-hypersensitivity phenotype of *ein3eil1*. Toward this end, we generated the *35S:SIED1/ein3eil1* transgenic plants. Compared with *ein3eil1*, the seedlings of *35S:SIED1/ein3eil1* showed significantly increased survival rate and fresh weight upon salt treatment (Figure 5H, 5I), indicating that SIED1 acts genetically downstream of EIN3 and overexpression of *SIED1* is sufficient to suppress the salt-hypersensitivity phenotype of *ein3eil1*. As expected, overexpression of *SIED1* also repressed the salt-hypersensitivity phenotype of *sied1* mutant (Figure 5H, 5I). Taken together, these results identify *SIED1*, acting downstream of EIN3, as a novel component that plays a positive role in salt tolerance.

### EIN3 Induces the Transcription of Peroxidases (POD) and Increases POD Activity and Diminishes ROS Accumulation

**Figure 6:**
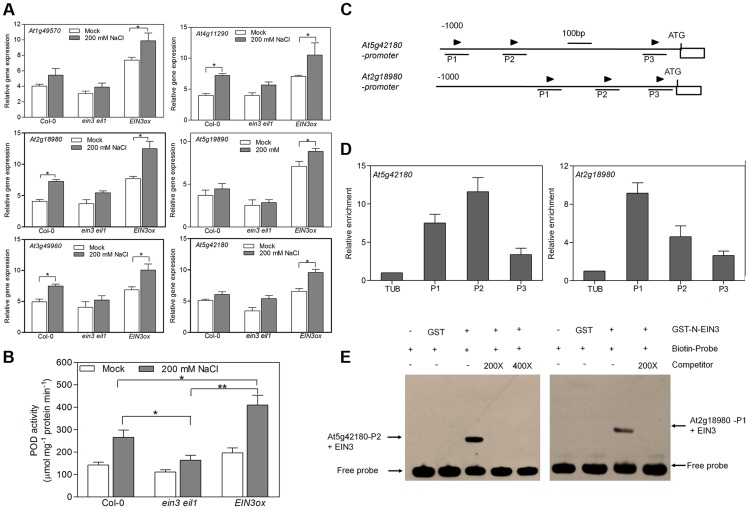
EIN3 increases activity of peroxidases through transcriptional regulation of POD genes directly. (A) qRT-PCR analysis of *POD*s expression. (B) Measurement of POD activity. The treated seedlings in (A) were also used (Student's *t* test, *P<0.05 and **P<0.01). (C) Schematic diagrams of putative EIN3 Binding Site (EBS) (arrows) in the promoters of two *POD* genes. The 1 kb upstream sequences are shown, and the translational start sites (ATG) are shown at position +1. (D) ChIP-qPCR assays of the promoter regions of *POD* genes from DNA of Col-0 seedlings with anti-EIN3 antibody. A Tubulin 8 fragment was amplified as control. Three biological replicates and two technical replicates were performed with similar results. (E) EMSA showing the interaction between the EBS containing region of *POD* genes and EIN3 protein. GST-tagged EIN3 N-terminus fusion protein was incubated with biotin-labeled DNA fragment. Competition for the biotin-labeled promoter region was done by adding an excess of unlabeled wild-type probe (competitor) or mutated probe (mutant competitor). Two biological replicates and two technical replicates were performed with similar results.

The transcriptome profiling analysis revealed that genes encoding oxidoreductase activity are enriched in SIED, suggesting that EIN3 might modulate the oxidative/reductive status under salt stress condition. Interestingly, we found that the expression of many genes encoding peroxidases (PODs) was induced in wild type by salt treatment, whose expression was also elevated in *EIN3ox* but reduced in *ein3eil1* under salt stress (Table S1 and Figure S12). These observations were further confirmed by qRT-PCR results with six selected *POD* genes (Figure 6A). In the meanwhile, we did not find evident differences in the expression levels of genes encoding superoxide dismutases (SOD), catalases (CAT1, CAT2 and CAT3) and NADPH oxidases (AtRobhA-F) among the three genotypes upon salt treatment (Figure S13A–C). These results suggest that EIN3 selectively induces the expression of genes encoding peroxidases. Peroxidase activity assay also showed that POD activity was significantly higher in *EIN3ox* seedlings than that in wild type (P<0.05) or *ein3eil1* (P<0.01) under salt stress condition (Figure 6B).

We next analyzed the promoter regions of two selected *POD* genes (At5g42180 and At2g18980) and found three EBSs in each promoter (Figure 6C). Chromatin immunoprecipitation (ChIP) assay using wild-type seedlings showed that the anti-EIN3 antibody bound strongly to the P2 fragment of At5g42180 and the P1 fragment of At2g18980 (Figure 6D) respectively, suggesting that EIN3 binds directly to the promoter regions of these genes *in vivo*. Furthermore, the promoter sequences of At5g42180 (P2) and At2g18980 (P1) were used for electrophoresis mobility shift assay (EMSA), showing that EIN3 can bind to these promoter sequences *in vitro* (Figure 6E). These results indicate that EIN3 increases peroxidase activity through the direct transcriptional regulation of *POD*s expression.

**Figure 7:**
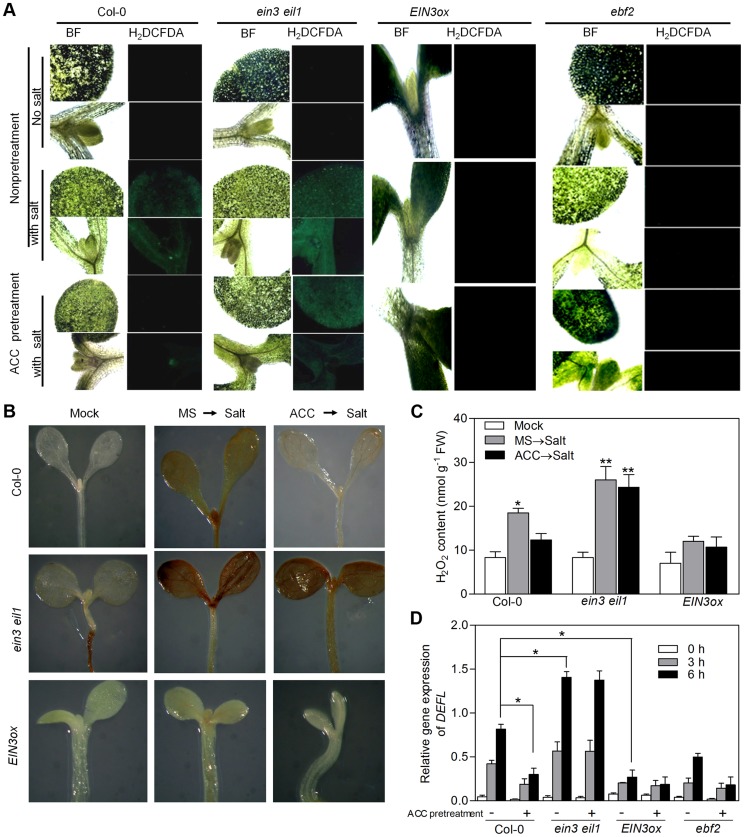
ROS accumulation in salt-treated Col-0, *ein3eil1*, *EIN3ox* and *ebf2* plants. (A) Fluorescence microscopy images of ROS (indicated by H_2_DCFDA fluorescence). Seedlings grown on MS medium supplemented with or without 10 µM ACC for 5 d were subjected to salt treatment. BF: bright field. Experiments were repeated three times with similar results. (B) DAB staining of seedlings under normal conditions or salt treatment. Seedlings grown on MS medium supplemented with or without 10 µM ACC for 5 d were treated with 200 mM NaCl for 6 h, and used for DAB staining. (C) H_2_O_2_ content in the seedlings in (B) (Student's *t* test, *P<0.05 and **P<0.01). (D) qRT-PCR analysis of *DEFL* expression (a ROS marker gene) (Student's *t* test, *P<0.05 and **P<0.01).

Peroxidases have been shown to participate in plant response against abiotic stresses as key scavengers of ROS [41]. The induction of several *POD* genes by EIN3 suggested that activation of EIN3 might facilitate the scavenging of ROS when plants are stressed with high salinity, thus leading to enhanced salt tolerance. To test this possibility, we determined the endogenous ROS accumulation and H_2_O_2_ content of wild-type Col-0, *ein3eil1* and *EIN3ox* upon salt treatment. Salt treatment evidently increased ROS accumulation and H_2_O_2_ content in the cotyledons of Col-0, indicated by H_2_DCFA fluorescence [42], [43] and DAB (3,3-diaminobenzidine) staining [44], respectively (Figure 7A–C). We further found higher level of ROS and H_2_O_2_ accumulation in *ein3eil1* while lower level of ROS and H_2_O_2_ production in *EIN3ox* than that of Col-0 upon salt treatment (Figure 7A–C), in accordance with the salt tolerance phenotypes of these genotypes (Figure 1). Additionally, we found that upon salt treatment, a higher ROS accumulation (indicated by DAB staining and H_2_O_2_ contents) was found in *sied1* mutant plants, while a significant decrease was observed in *SIED1ox* seedlings (Figure S14), suggesting that SIED1 acts to enhance salt tolerance also via reducing ROS accumulation.

Since ACC-pretreated plants exhibited tolerance to salt stress, we next examined the effect of ACC pretreatment on ROS accumulation and H_2_O_2_ production in salt-treated plants. Compared with non-pretreated seedlings, ACC-pretreated Col-0 showed evident lower levels of ROS accumulation and H_2_O_2_ generation when stressed with high salinity (Figure 7A–C). Meanwhile, ROS level and H_2_O_2_ content remained constantly high in *ein3eil1* and low in *EIN3ox*, and no obvious changes were found in these two genotypes upon ACC pretreatment (Figure 7A–C). These results are also in good correlation with their respective salt stress phenotypes with or without ACC pretreatment (Figure 1). We next examined the expression levels of a well-established ROS marker gene, *DEFL* (defensin-like), which was shown to be induced by various ROS [45]. In agreement with the histochemical observations of ROS accumulation, ACC-pretreated Col-0 seedlings accumulated less *DEFL* mRNA than non-pretreated Col-0 upon 3 h and 6 h of salt applications (Figure 7D). By contrast, *DEFL* expression was highly induced by salt stress in *ein3 eil1* regardless of ACC pretreatment, whereas its expression levels remained constantly lower in salt-stressed *EIN3ox* and *ebf2-1* than that in Col-0 (Figure 7D), further supporting the importance of EIN3 action in the modulation of salt-evoked ROS accumulation.

Taken together, our results indicate that activation of EIN3, either by *EIN3* overexpression, ACC pretreatment, or *EBF2* mutation, induces the expression of numerous *POD* and *SIED* genes, which arguably contributes to the decreased accumulation of ROS, and consequently the detoxification of salt stress-elicited damages.

## Discussion

### Salt Stress Stabilizes EIN3/EIL1 Proteins and Destabilizes EBF1/EBF2 Proteins in Both EIN2-Dependent and EIN2-Independent Manners

Genetic and biochemical studies revealed that EIN3/EIL1 proteolysis is mediated by two F-box proteins, EBF1/EBF2. Upon ethylene treatment, the levels of EBF1/EBF2 proteins are down-regulated through 26S proteasome pathway, which leads to the accumulation of EIN3 and EIL1 proteins [16], [18], [19], [20]. Moreover, our previous work indicated that EIN2 is indispensable for ethylene-induced EIN3 and EIL1 stabilization and degradation of EBF1/EBF2 proteins, because no EIN3 or EIL1 protein can be detected in *ein2* but EBF1/EBF2 proteins are constitutively accumulated in the *ein2* background [18]. In this study, we found that the level of EIN3 and EIL1 proteins was remarkably up-regulated by salt treatment in wild type, and also elevated in *ein2-5* background, although to a lesser extent. Conversely, we further found that the levels of EBF1/EBF2 proteins were evidently down-regulated by salt treatment in both Col-0 and *ein2-5* background, which is the result of 26S proteasome-executed EBF1/EBF2 degradation. These findings indicate that EIN2 is dispensable for salt-induced EIN3/EIL1 accumulation and EBF1/EBF2 degradation, which is distinct from the regulatory mechanism in ethylene signaling that fully depends on EIN2. Thus, we propose that salt treatment promotes EBF1/EBF2 protein degradation, which consequently induces EIN3 protein accumulation in both EIN2-dependent and EIN2-independent pathways.

These findings for the first time report the existence of an alternative pathway that is distinct from the canonical ethylene signaling pathway to modulate the protein stability of EBF1/EBF2 and EIN3/EIL1. In addition to NaCl, we found that treatments with equal concentration of KCl, NaNO_3_ or KNO_3_ salt also effectively induced EBF2-GFP protein degradation in *ein2-5* mutant (Figure S7A), suggesting that this regulation is a general salt stress response rather than the specific effect caused by sodium chloride. High salt condition often affects osmotic homeostasis and causes osmotic stresses. However, treatment with 200 mM mannitol did not affect EBF2-GFP protein level, suggesting that the ionic stress but not osmotic stress regulates EBF1/EBF2 protein turnover (Figure S7B). Further investigation is needed to elucidate the regulatory mechanism behind the salt-induced proteasomal degradation of EBF1/EBF2.

In this study, we demonstrate that the protein stability of EBF1/EBF2 and EIN3/EIL1 is regulated by ethylene and salt stress in different ways. Recently, increasing body of evidence indicates that EIN3/EIL1 might act as a signaling hub that integrates multiple hormone and stress signals [18], [31], [43], [46], [47], [48], [49], [50]. Our previous works and other studies reported that EIN3 protein stability is also controlled by light irradiation [43], auxin and its biosynthesis inhibitor, Kyn [28], and glucose [51]. These studies collectively indicate that EIN3/EIL1 are not limited to the ethylene signaling, but rather participating in the regulation of myriad processes, whose function and/or abundance are also modulated by various signals besides the ethylene gas. Given that only a subset of abiotic stresses (e.g. high salt, high sucrose, freezing, but not osmosis) alter the stability of EIN3 or EBF proteins, it is thus interesting to ascertain whether, and if so, how other types of abiotic stresses, such as drought, chilling, heat, and heavy metals, affect the stabilization of EBF1/EBF2 and EIN3/EIL1 proteins. The elucidation of differential regulation of EIN3 and/or EBF protein stability by environmental and stress signals would provide insights into how EIN3 exerts its unique effect in plant adaptation to various growth conditions.

### EIN3 Enhances Salt Tolerance by Decreasing ROS Accumulation

Previous studies revealed a number of salt-tolerance genes that might be EIN3 target genes, such as *ESE1* (an AP2/EREBP transcription factor) [52] and *JERF3* [53]. However, a systematic analysis of EIN3-regulated genes in salt tolerance is lacking. In this study, we conducted a genome-wide transcriptome profiling in combination with genetic approach to dissect and identify numerous EIN3-regulated genes and pathways that might contribute to ethylene-directed salt tolerance (Table S1).

One of the pathways is the scavengers of ROS, in which EIN3 up-regulates the expression of numerous peroxidases. One important cause of high salinity-imposed damage is ROS generated by salt stress. ROS plays a dual role in plants, as actors or modulators of cellular signaling pathways on one hand, and as oxidative agents or toxic products elicited by cellular stresses on the other hand [54]. ROS is tightly regulated by the equilibrium between production and scavenging. Transgenic plants overexpressing enzymes involved in oxidative protection, such as glutathione peroxidase (GPX) [55], superoxide dismutase (SOD) [56], ascorbate peroxidase (APX) [57], exhibited enhanced salt tolerance. In this study, based on the transcriptome analysis, we found that the expression of several ROS scavenger peroxidases (PODs) was notably elevated in *EIN3ox* under salt condition compared with that in wild type or *ein3 eil1* (Table S1 and Figure 6). Accordingly, the overall activity of POD enzymes was significantly higher in *EIN3ox* than that in wild type or *ein3 eil1* upon salt stress (Figure 6B). The transcript level of a zinc-finger transcription factor, ZAT12, which has been shown to induce the expression of *APX1* [35], was also up-regulated in *EIN3ox* (Table S1). Consistently, we observed lower levels of H_2_O_2_ accumulation and ROS marker gene expression in *EIN3ox* but higher levels in *ein3 eil1* compared with wild type upon salt treatment (Figure 7). Thus, elimination of excessive ROS accumulation under salt stress through inducing the expression of peroxidases is one contributing mechanism behind EIN3-mediated salt tolerance.

In addition, we found that EIN3/EIL1 enhanced salt tolerance through regulating a myriad of *SIED* genes. We provided genetic evidence to indicate that a portion of these *SIED* genes participate in salt tolerance, including 5 genes previously known to be induced by and/or involved in various abiotic stresses, and a novel gene (*SIED1*) whose function is previously unknown. Further biochemical studies uncovered that 5 of these 6 *SIED* genes, including *SIED1*, are direct target genes of EIN3. We found that overexpression of *SIED1* also decreased ROS accumulation upon salt treatment (Figure S14). Nevertheless, the lack of salt stress phenotype for other *SIED* knockout mutants does not necessarily mean that these genes are not involved in salt tolerance. For instance, it is well known that ERF family transcription factors and JAZ family transcriptional regulators possess tremendous functional redundancy within the family members [31], [58], [59]. In addition, we cannot completely rule out the possibility that the lack of salt stress phenotype is simply because the T-DNA insertions in many *SIED* genes may have a marginal effect on gene expression/function (Table S4). Additional mutant alleles, gain-of-function studies or multigenic mutants analysis would help clarify whether those *SIED* genes play a role in EIN3-induced salt tolerance. Based on our genomic and genetic studies, the identification of numerous *SIED* and *SRED* genes would thus serve as a proper starting point to further dissect the complicated signaling network that is directed by EIN3/EIL1 in plant adaptation to salt stress.

Intracellular K^+^/Na^+^ homeostasis is crucial for cell metabolism and is considered to be a key component of salinity tolerance in plants. Jiang et al. recently reported that salinity-induced ethylene is a potent promoter of salt tolerance through enhancing Na^+^/K^+^ homeostasis in *Arabidopsis* [25]. In fact, we also found that salt-stressed *EIN3ox* seedlings had accumulated more K^+^ and less Na^+^, whereas *ein3-1 eil1-1* seedlings had higher Na^+^ but lower K^+^ content compared with wild type (data not shown). These results indicate that the modulation of intracellular K^+^/Na^+^ homeostasis serves as another contributing mechanism for ethylene-induced tolerance to high salt stress.

In summary, our study provides new insights into how ethylene enhances plants' tolerance to high salinity. We propose that EIN3 and EIL1 play a central role in conferring salt tolerance via at least two mechanisms, one is to modulate intracellular K^+^/Na^+^ homeostasis, the other is to deter ROS accumulation by inducing *SIED*s and *POD*s gene expression. Our study also provides a possible explanation for the priming effect of ethylene pretreatment, as ethylene treatment decreases EBF1/2 stability and increases EIN3/EIL1 abundance, which enhances the sensitivity of this pathway to salt stress signal. Once the EIN3 pathway is activated in advance (by ethylene/ACC), it will alter the expression of downstream *SIED* genes, many of which are direct target genes of EIN3, and induce a myriad of defense pathways, and switch plants to a more resistant state.

## Materials and Methods

### Plant Material, Growth Conditions and Salt Stress Experiments

*Arabidopsis thaliana* ecotype Col-0 was the parent strain for all mutants and transgenic lines used in this study. Surface-sterilized seeds were plated on MS medium supplemented with or without 10 µm ACC and imbibed for 4 d in 4°C to improve germination uniformity. For phenotypic analysis under salt stress, seedlings were transferred onto MS agar plates containing 200 mM NaCl and their subsequent appearance was recorded photographically 3 days after transfer. The salt stress of seedlings was indicated by visibly bleached leaves.

### Measurements of ROS, Relative Electrolyte Leakage and Chlorophyll Content

Reactive oxygen species (ROS) accumulation in seedlings was detected using the cell-permeable fluorescent probe 2′,7′-dichlorodihydrofluorescein diacetate (DCFH2-DA; Molecular Probes) according to Schopher et al. [42]. 5-day-old seedlings grown on MS with or without 10 µM ACC were treated with 200 mM NaCl for 6 h. Then, seedling were incubated in 100 µM DCFH2-DA in 1% ethanol for 20 min, and washed with distilled H_2_O to remove the dye before the observation of ROS accumulation under the confocal microscope. Confocal images were obtained after excitation at 488 nm and emission at 522 nm. H_2_O_2_ production was detected in seedlings using 3, 3-diaminobenzidine (DAB) as substrate. Relative electrolyte leakage and chlorophyll content were measured as described previously [60].

### Assay for GUS Activity

Histochemical and Fluorimetric GUS assays were performed using the method described by Jefferson [61].

### Confocal Laser Microscopy

A Leica TCS SP2 inverted confocal laser microscope with ×40 objectives was used to detect GFP fluorescence. The excitation wavelength was 488 nm, and a bandpath filter of 510 to 525 nm was used for emission.

### Protein Extract and Western Blotting

Plant samples were ground in liquid N_2_ and soluble protein extracts were made by homogenization in 50 mM Tris–HCl (pH 8.0), 10 mM NaCl, 0.1 M PMSF, and 0.1 M DTT, with subsequent centrifugation at 13.000× g for 30 min at 4°C. The protein in the supernatants was quantified by Bradford's assay (Bradford, 1976). Western blot analysis was performed as described previously [18], [60] with anti-GFP (Invitrogen), anti-FLAG (Sigma-Aldrich), anti-MYC (AbChem), or anti-EIN3 antibodies [16].

### qRT-PCR Assay

Total RNA was extracted from seedlings and analyzed as described previously [62]. First strand cDNA samples were generated from total RNA samples by reverse transcription using an AMV reverse transcriptase 1^st^ strand cDNA synthesis kit (Life Sciences, Promega) and were used as templates for qPCR-based gene expression analysis as described previously [60]. The oligonucleotide primer sequences used to amplify specific cDNAs were described in Table S5.

### Whole-Genome Transcriptome Profiling Analysis

Microarray experiments were performed using *Arabidopsis* Affymetrix chips (Santa Clara, CA). Total RNA was extracted from 5-d-old post germinated seedlings grown on MS medium, which were subsequently subjected to 200 mM NaCl for 6 h. Each experiment used two biological replicates, and each represented a pool of around 200 seedlings from two individual plates. The expression data were analyzed using Gene Spring version 4.2.1 (Silicon Genetics Inc., Red- wood City, CA, USA). A q value<0.05 and fold change >2 between control and treatment samples were considered as a cutoff.

### Gene Ontology (GO) Enrichment Analysis

GO enrichment analysis on SIED genes was performed using the software GOrilla (**G**ene **O**ntology en**RI**chmentana**L**ysis and visua**L**iz**A**tion tool) as described previously [33].

### ChIP and EMSA Assays

10 g of 5-d-old Col-0 seedlings were prepared for ChIP assays using anti-EIN3 antibody [16], and the enriched DNA fragments were measured by qPCR as previously described [60]. All assays were performed with two biological replicates and three technical replicates. EMSA assay was performed as described previously [60]. The N-terminus DNA binding domain of EIN3 protein (amino acids 141 to 352) that is sufficient for DNA binding [31] was expressed as a glutathione *S*-transferase (GST) fusion protein in *Escherichia coli* and purified, and used for EMSA experiments.

### Generation of Transgenic Plants

Plants overexpressing *SIED1* were generated by *Agrobacterium tumefaciens* strain GV3101 -mediated transformation into *Arabidopsis* Col-0 by floral dip [63], using a construct that contained the full-length coding region of *SIED1* (At5g22270) in the PBI121vector. To generate *pSIED1:GUS* construct, a 2.2-kb *SIED1* promoter region was amplified from genomic DNA and inserted into PBI101 vector, introduced into GV3101, and transformed into Col-0, *ein3 eil1* and *EIN3ox* plants [63]. To generate *EIL1pro:EIL1-GFP* construct, the promoter region and the full-length coding region of *EIL1* was amplified from genomic DNA and inserted into pCHF3 vector, introduced into GV3101, and transformed into wild-type Col-0 and *ein2-5* plants.

### Statistical Analysis

The values we obtained in the figures were expressed as the means (SD). Two-tailed Student's *t* tests were used.

### Accession Numbers

The microarray data reported in this paper have been deposited at NASC (The Nottingham Arabidopsis Stock Centre) database under accession number NASCARRAYS-659.

## Supporting Information

Figure S1 Overexpression of *EIL1* increases salt tolerance. (A) Seedlings were grown on MS medium for 5 d and then transferred onto MS medium with 0, 50, 100, 150, 200 mM NaCl for 7 d. (B) Survival rate and (C) relative root elongation of seedlings shown in (A). Seedling death was scored as complete bleaching of cotyledons and leaves. Root length of seedlings transferred to MS medium without salt was set to 100%. Values are mean ± SD from 30 seedlings per replicate (*n* = 3 replicates). (Student's *t* test, *P<0.05 and **P<0.01).
(TIF)

Figure S2 ACC pretreatment or enhanced ethylene signaling increases salt tolerance. (A) Seedlings were grown on MS medium with or without 10 µM ACC for 5 d and then transferred onto MS medium with 0, 50, 100, 150, 200 mM NaCl for 7 d. (B) and (E) Survival rate of seedlings shown in (A). Seedlings grown on MS (B) or ACC (E) were transferred to MS medium supplemented with NaCl. Seedling death was scored as complete bleaching of cotyledons and leaves. Values are mean ± SD from 30 seedlings per replicate (*n* = 3 replicates). (Student's *t* test, *P<0.05 and **P<0.01). (C) and (F) Relative fresh weight of seedlings shown in (A). Seedlings grown on MS (C) or ACC (F) were transferred to MS medium supplemented with NaCl. Fresh weight of seedlings transferred to MS medium without salt was set to 100%. Values are mean ± SD from 30 seedlings per replicate (*n* = 3 replicates). (Student's *t* test, *P<0.05 and **P<0.01). (D) and (G) Relative root elongation of seedlings shown in (A). Seedlings grown on MS (D) or ACC (G) were transferred to MS medium supplemented with NaCl. Root length of seedlings transferred to MS medium without salt was set to 100%. Values are mean ± SD from 30 seedlings per replicate (*n* = 3 replicates). (Student's *t* test, *P<0.05 and **P<0.01).
(TIF)

Figure S3 Inducible overexpression of *EIN3* is sufficient to confer salt tolerance. (A) Western blot analysis of EIN3-FLAG protein accumulation. β-estradiol-inducible EIN3-FLAG in the *ein3 eil1 ebf1 ebf2* background (*iE/qm*) seedlings grown on MS for 5 d and pretreated with 100 µM β-estradiol for 2 h. After washing with water for 3 times, the seedlings were then treated with 100 µM ACC or 200 mM NaCl for another 3 h or 6 h. Proteins were extracted and subjected to immunoblots using anti-FLAG antibody. Experiments were repeated three times with similar results. (B) Plants were grown on MS medium for 5 d and then transferred onto MS medium supplemented with (+) or without (−) 200 mM NaCl in the presence of indicated concentrations of estradiol for 3 d. (C) Survival rate of plants shown in (B). Values are mean ± SD from 30 seedlings per replicate (*n* = 3 replicates). (Student's *t* test, *P<0.05 and **P<0.01). (D) to (F) Quantification of total chlorophyll content (D), relative electrolyte leakage (E) and fresh weights (F) of *iE/qm* seedlings grown on MS medium supplemented with or without 200 mM NaCl and 1 µM β-estradiol for 3 d. Values are mean ± SD from 25 seedlings per replicate (*n* = 5 replicates). (Student's *t* test, *P<0.05 and **P<0.01).
(TIF)

Figure S4 Salt treatment did not alter *EIN3* mRNA level. 5-d-old seedlings grown on MS medium were transferred into liquid culture medium with or without 200 mM NaCl for 6 h. Total RNA was extracted and subjected to northern blots analysis with labeled *EIN3* cDNA probes. Ethidium bromide staining of the gel was shown in the bottom image as loading controls.
(TIF)

Figure S5 Western blot analyses of EIN3 protein accumulation in mock-treated Col-0 and *ein2-5* for 6 h, and in salt-treated *etr1-1* for 3 h and 6 h. (A) EIN3 protein accumulation in mock-treated Col-0 and *ein2-5*. 5-d-old Col-0 or *ein2-5* seedlings grown on MS medium were transferred into liquid culture medium for 6 h. Total protein was extracted and subjected to Western blot analysis with anti-EIN3 antibody. A nonspecific band was used as a loading control. Experiments were repeated three times with similar results. (B) EIN3 protein accumulation in salt-treated *etr1-1*. 5-d-old *etr1-1* seedlings were treated with 200 mM NaCl for 3 h and 6 h. Protein was extracted and subjected to immunoblots using anti-EIN3 antibody. A nonspecific band was used as a loading control.
(TIF)

Figure S6 Salt treatment promotes protein accumulation of EIL1 in both EIN2-dependent and EIN2-independent manners. 5-d-old seedlings of *pEIL1-EIL1-GFP*/*ein2-5* and *pEIL1-EIL1-GFP*/Col-0 were treated with 200 mM NaCl for 3 h and 6 h. 5-d-old Col-0 seedlings treated with 200 mM NaCl for 6 h were used as the negative control. Protein was extracted and subjected to immunoblots using anti-GFP antibody. A nonspecific band was used as a loading control.
(TIF)

Figure S7 Ionic stresses but not osmotic stress regulate EBF2-GFP protein stability in an EIN2 independent manner. (A) Immunoblot assay of EBF2-GFP protein in *ein2-5* background upon treatment with NaCl, KCl, NaNO_3_ and KNO_3_. Transgenic seedlings grown on MS medium for 5 d were subjected to 200 mM NaCl, 200 mM KCl, 200 mM NaNO_3_ or 200 mM KNO_3_ for 3 h and 6 h. Protein levels of EBF2-GFP were analyzed by Western blot using an anti-GFP antibody. Experiments were repeated three times with similar results. (B) Immunoblot assay of EBF2-GFP protein in *ein2-5* background upon treatment with mannitol. Transgenic seedlings grown on medium for 5 d were subjected to 200 mM mannitol for indicated time. Protein levels of EBF2-GFP were analyzed by Western blot using an anti-GFP antibody. Experiments were repeated three times with similar results.
(TIF)

Figure S8 The enriched GO terms in *SIED* genes. (A and B) The network graphs show Gorilla visualization of GO terms for *SIED* genes: biological process (A) and molecular function (B). Colored nodes represent GO terms that are significantly overrepresented (P value<0.05), with the shade indicating significance as shown in the color bar. A more detailed analysis of the GO categories is shown in Table S1 online.
(TIF)

Figure S9 qRT-PCR analysis of selected EIN3-induced genes. (A–F) qRT-PCR analysis of selected EIN3-induced genes in Col-0, *ein3eil1* and *EIN3ox* seedlings. 5-d-old seedlings grown on MS medium were transferred into liquid culture medium with or without 200 mM NaCl for 6 h. Total RNA was extracted and subjected to qRT-PCR analysis. Three biological replicates and two technical replicates were performed.
(TIF)

Figure S10 PCR genotyping of the Salk T-DNA mutants of the six *SIED* genes. 5-d-old seedlings were subjected to DNA extraction and subsequent PCR analysis. G, gene specific primers; T, the T-DNA left border primer used in combination with gene specific primers.
(TIF)

Figure S11 EIN3 protein directly binds to the promoters of several *SIED* genes. (A) Schematic diagrams of putative EIN3 Binding Site (EBS) (arrows) in the promoters of six *SIED* genes and DNA fragments (P1, P2, P3 and P4) used for ChIP or EMSA experiments. The 1.8 or 2.0 kb upstream sequences are shown, and the translational start site (ATG) is shown at position +1. (B)–(G) Chromatin immunoprecipitation (ChIP)-qPCR assays of the promoter regions of SIED genes from DNA of Col-0 seedlings with anti-EIN3 antibody. A Tubulin 8 fragment was amplified as control. Three biological replicates and two technique replicates were performed with similar results. (H)–(K) EMSA showing the interaction between the EBS containing region of *SIED* genes and EIN3 protein. GST-tagged EIN3 N-terminus (aa 141–352) fusion protein was incubated with biotin-labeled DNA fragment. Competition for the biotin-labeled promoter region was done by adding an excess of unlabeled wild-type probe (competitor) or mutated probe (mutant competitor). Two biological replicates and two technique replicates were performed with similar results.
(TIF)

Figure S12 Transcriptome profiling of genes encoding PODs in Col-0, *ein3 eil1* and *EIN3ox* (A–C). Transcriptome profiling and data analysis were performed as described in “Methods”. Genes Exhibiting higher expression level in *EIN3ox* plants were highlighted with arrows.
(TIF)

Figure S13 Transcriptome profiling of genes encoding Superoxide Dismutase (SOD) (A), Catalase (CAT1-3) (B) and NADPH Oxidase (RobhA-F) (C) in Col-0, *ein3 eil1* and *EIN3ox*.
(TIF)

Figure S14 ROS accumulation in salt-treated Col-0, *sied1* and *35S:SIED1* plants. (A) DAB staining of seedlings under normal conditions or salt treatment. Seedlings grown on MS medium for 5 d were treated with or without 200 mM NaCl for 6 h, and used for DAB staining. Experiments were repeated three times with similar results. (B) H_2_O_2_ content in the seedlings in (A) (Student's *t* test, *P<0.05 and **P<0.01).
(TIF)

Figure S15 Na^+^ and K^+^ Content in Salt-treated Col-0, *ein3eil1* and *EIN3ox* plants. 5-d-old seedlings grown on MS medium were transferred into liquid culture medium with or without 200 mM NaCl for 6 h. Na^+^ content (A), K^+^ content (B) and K^+^ / Na^+^ ration (C) in the whole seedlings were examined. Values are mean ± SD (*n*  =  3 replicates). dw, dry weight.
(TIF)

Table S1 Salt-Induced EIN3/EIL1-Dependent (SIED) genes (114). Genes highlighted in red are direct targets of EIN3 that were identified by ChIP-Seq experiments.
(DOC)

Table S2 Salt-Repressed EIN3/EIL1-Dependent (SRED) genes (14).
(DOC)

Table S3 Genes up-regulated more than 5-fold in *EIN3ox* versus Col-0 under normal condition (62). Genes up-regulated more than 2-fold in salt-treated Col-0 were highlighted in red.
(DOC)

Table S4 Summary of genes, Salk T-DNA lines, insertion site, description and phenotype on salt medium. Numbers in the brackets are the exact positions of T-DNA insertion, and minus indicates that the insertion site is located in the upstream of coding region. Mutants exhibiting hypersensitivity to salt stress were highlighted in red.
(DOC)

Table S5 Primers used in this work.
(DOC)

## References

[1] Apse MP, Aharon GS, Snedden WA, Blumwald E (1999) Salt tolerance conferred by overexpression of a vacuolar Na+/H+ antiport in Arabidopsis. Science 285: 1256–1258. 10455050 10.1126/science.285.5431.1256

[2] Bradford KJ, Yang SF (1980) Stress-induced Ethylene Production in the Ethylene-requiring Tomato Mutant Diageotropica. Plant Physiol 65: 327–330. 16661183 10.1104/pp.65.2.327 PMC440320

[3] Bleecker AB, Kende H (2000) Ethylene: a gaseous signal molecule in plants. Annu Rev Cell Dev Biol 16: 1–18. 11031228 10.1146/annurev.cellbio.16.1.1

[4] Ecker JR (1995) The ethylene signal transduction pathway in plants. Science 268: 667–675. 7732375 10.1126/science.7732375

[5] Chen YF, Etheridge N, Schaller GE (2005) Ethylene signal transduction. Ann Bot 95: 901–915. 15753119 10.1093/aob/mci100 PMC4246747

[6] Chen YF, Randlett MD, Findell JL, Schaller GE (2002) Localization of the ethylene receptor ETR1 to the endoplasmic reticulum of Arabidopsis. J Biol Chem 277: 19861–19866. 11916973 10.1074/jbc.M201286200

[7] Chang C, Kwok SF, Bleecker AB, Meyerowitz EM (1993) Arabidopsis ethylene-response gene ETR1: similarity of product to two-component regulators. Science 262: 539–544. 8211181 10.1126/science.8211181

[8] Hua J, Chang C, Sun Q, Meyerowitz EM (1995) Ethylene insensitivity conferred by Arabidopsis ERS gene. Science 269: 1712–1714. 7569898 10.1126/science.7569898

[9] Hua J, Meyerowitz EM (1998) Ethylene responses are negatively regulated by a receptor gene family in Arabidopsis thaliana. Cell 94: 261–271. 9695954 10.1016/s0092-8674(00)81425-7

[10] Kieber JJ, Rothenberg M, Roman G, Feldmann KA, Ecker JR (1993) CTR1, a negative regulator of the ethylene response pathway in Arabidopsis, encodes a member of the raf family of protein kinases. Cell 72: 427–441. 8431946 10.1016/0092-8674(93)90119-b

[11] Alonso JM, Hirayama T, Roman G, Nourizadeh S, Ecker JR (1999) EIN2, a bifunctional transducer of ethylene and stress responses in Arabidopsis. Science 284: 2148–2152. 10381874 10.1126/science.284.5423.2148

[12] Bisson MM, Bleckmann A, Allekotte S, Groth G (2009) EIN2, the central regulator of ethylene signalling, is localized at the ER membrane where it interacts with the ethylene receptor ETR1. Biochem J 424: 1–6. 19769567 10.1042/BJ20091102

[13] Ju C, Yoon GM, Shemansky JM, Lin DY, Ying ZI, et al (2012) CTR1 phosphorylates the central regulator EIN2 to control ethylene hormone signaling from the ER membrane to the nucleus in Arabidopsis. Proc Natl Acad Sci U S A 109: 19486–19491. 23132950 10.1073/pnas.1214848109 PMC3511113

[14] Qiao H, Shen Z, Huang SS, Schmitz RJ, Urich MA, et al (2012) Processing and subcellular trafficking of ER-tethered EIN2 control response to ethylene gas. Science 338: 390–393. 22936567 10.1126/science.1225974 PMC3523706

[15] Wen X, Zhang C, Ji Y, Zhao Q, He W, et al (2012) Activation of ethylene signaling is mediated by nuclear translocation of the cleaved EIN2 carboxyl terminus. Cell Res 22: 1613–1616. 23070300 10.1038/cr.2012.145 PMC3494400

[16] Guo H, Ecker JR (2003) Plant responses to ethylene gas are mediated by SCF(EBF1/EBF2)-dependent proteolysis of EIN3 transcription factor. Cell 115: 667–677. 14675532 10.1016/s0092-8674(03)00969-3

[17] Solano R, Stepanova A, Chao Q, Ecker JR (1998) Nuclear events in ethylene signaling: a transcriptional cascade mediated by ETHYLENE-INSENSITIVE3 and ETHYLENE-RESPONSE-FACTOR1. Genes Dev 12: 3703–3714. 9851977 10.1101/gad.12.23.3703 PMC317251

[18] An F, Zhao Q, Ji Y, Li W, Jiang Z, et al (2010) Ethylene-induced stabilization of ETHYLENE INSENSITIVE3 and EIN3-LIKE1 is mediated by proteasomal degradation of EIN3 binding F-box 1 and 2 that requires EIN2 in Arabidopsis. Plant Cell 22: 2384–2401. 20647342 10.1105/tpc.110.076588 PMC2929093

[19] Gagne JM, Smalle J, Gingerich DJ, Walker JM, Yoo SD, et al (2004) Arabidopsis EIN3-binding F-box 1 and 2 form ubiquitin-protein ligases that repress ethylene action and promote growth by directing EIN3 degradation. Proc Natl Acad Sci U S A 101: 6803–6808. 15090654 10.1073/pnas.0401698101 PMC404126

[20] Potuschak T, Lechner E, Parmentier Y, Yanagisawa S, Grava S, et al (2003) EIN3-dependent regulation of plant ethylene hormone signaling by two arabidopsis F box proteins: EBF1 and EBF2. Cell 115: 679–689. 14675533 10.1016/s0092-8674(03)00968-1

[21] van Loon LC, Geraats BP, Linthorst HJ (2006) Ethylene as a modulator of disease resistance in plants. Trends Plant Sci 11: 184–191. 16531096 10.1016/j.tplants.2006.02.005

[22] Achard P, Cheng H, De Grauwe L, Decat J, Schoutteten H, et al (2006) Integration of plant responses to environmentally activated phytohormonal signals. Science 311: 91–94. 16400150 10.1126/science.1118642

[23] Cao WH, Liu J, He XJ, Mu RL, Zhou HL, et al (2007) Modulation of ethylene responses affects plant salt-stress responses. Plant Physiol 143: 707–719. 17189334 10.1104/pp.106.094292 PMC1803741

[24] Lei G, Shen M, Li ZG, Zhang B, Duan KX, et al (2011) EIN2 regulates salt stress response and interacts with a MA3 domain-containing protein ECIP1 in Arabidopsis. Plant Cell Environ 34: 1678–1692. 21631530 10.1111/j.1365-3040.2011.02363.x

[25] Jiang C, Belfield EJ, Cao Y, Smith JA, Harberd NP (2013) An Arabidopsis soil-salinity-tolerance mutation confers ethylene-mediated enhancement of sodium/potassium homeostasis. Plant Cell 25: 3535–3552. 24064768 10.1105/tpc.113.115659 PMC3809548

[26] Verslues PE, Agarwal M, Katiyar-Agarwal S, Zhu J, Zhu JK (2006) Methods and concepts in quantifying resistance to drought, salt and freezing, abiotic stresses that affect plant water status. Plant J 45: 523–539. 16441347 10.1111/j.1365-313X.2005.02593.x

[27] Stepanova AN, Yun J, Likhacheva AV, Alonso JM (2007) Multilevel interactions between ethylene and auxin in Arabidopsis roots. Plant Cell 19: 2169–2185. 17630276 10.1105/tpc.107.052068 PMC1955696

[28] He W, Brumos J, Li H, Ji Y, Ke M, et al (2011) A small-molecule screen identifies L-kynurenine as a competitive inhibitor of TAA1/TAR activity in ethylene-directed auxin biosynthesis and root growth in Arabidopsis. Plant Cell 23: 3944–3960. 22108404 10.1105/tpc.111.089029 PMC3246337

[29] Ismail A, Riemann M, Nick P (2012) The jasmonate pathway mediates salt tolerance in grapevines. J Exp Bot 63: 2127–2139. 22223808 10.1093/jxb/err426 PMC3295401

[30] Zhang H, Huang Z, Xie B, Chen Q, Tian X, et al (2004) The ethylene-, jasmonate-, abscisic acid- and NaCl-responsive tomato transcription factor JERF1 modulates expression of GCC box-containing genes and salt tolerance in tobacco. Planta 220: 262–270. 15300440 10.1007/s00425-004-1347-x

[31] Zhu Z, An F, Feng Y, Li P, Xue L, et al (2011) Derepression of ethylene-stabilized transcription factors (EIN3/EIL1) mediates jasmonate and ethylene signaling synergy in Arabidopsis. Proc Natl Acad Sci U S A 108: 12539–12544. 21737749 10.1073/pnas.1103959108 PMC3145709

[32] Chang KN, Zhong S, Weirauch MT, Hon G, Pelizzola M, et al (2013) Temporal transcriptional response to ethylene gas drives growth hormone cross-regulation in Arabidopsis. Elife 2: e00675. 23795294 10.7554/eLife.00675 PMC3679525

[33] Eden E, Navon R, Steinfeld I, Lipson D, Yakhini Z (2009) GOrilla: a tool for discovery and visualization of enriched GO terms in ranked gene lists. BMC Bioinformatics 10: 48. 19192299 10.1186/1471-2105-10-48 PMC2644678

[34] Davletova S, Schlauch K, Coutu J, Mittler R (2005) The zinc-finger protein Zat12 plays a central role in reactive oxygen and abiotic stress signaling in Arabidopsis. Plant Physiol 139: 847–856. 16183833 10.1104/pp.105.068254 PMC1256000

[35] Rizhsky L, Davletova S, Liang H, Mittler R (2004) The zinc finger protein Zat12 is required for cytosolic ascorbate peroxidase 1 expression during oxidative stress in Arabidopsis. J Biol Chem 279: 11736–11743. 14722088 10.1074/jbc.M313350200

[36] Vogel JT, Zarka DG, Van Buskirk HA, Fowler SG, Thomashow MF (2005) Roles of the CBF2 and ZAT12 transcription factors in configuring the low temperature transcriptome of Arabidopsis. Plant J 41: 195–211. 15634197 10.1111/j.1365-313X.2004.02288.x

[37] Sakamoto H, Maruyama K, Sakuma Y, Meshi T, Iwabuchi M, et al (2004) Arabidopsis Cys2/His2-type zinc-finger proteins function as transcription repressors under drought, cold, and high-salinity stress conditions. Plant Physiol 136: 2734–2746. 15333755 10.1104/pp.104.046599 PMC523337

[38] Sun J, Jiang H, Xu Y, Li H, Wu X, et al (2007) The CCCH-type zinc finger proteins AtSZF1 and AtSZF2 regulate salt stress responses in Arabidopsis. Plant Cell Physiol 48: 1148–1158. 17609218 10.1093/pcp/pcm088

[39] Sato T, Maekawa S, Yasuda S, Sonoda Y, Katoh E, et al (2009) CNI1/ATL31, a RING-type ubiquitin ligase that functions in the carbon/nitrogen response for growth phase transition in Arabidopsis seedlings. Plant J 60: 852–864. 19702666 10.1111/j.1365-313X.2009.04006.x

[40] Kreps JA, Wu Y, Chang HS, Zhu T, Wang X, et al (2002) Transcriptome changes for Arabidopsis in response to salt, osmotic, and cold stress. Plant Physiol 130: 2129–2141. 12481097 10.1104/pp.008532 PMC166725

[41] Hiraga S, Yamamoto K, Ito H, Sasaki K, Matsui H, et al (2000) Diverse expression profiles of 21 rice peroxidase genes. FEBS Lett 471: 245–250. 10767432 10.1016/s0014-5793(00)01409-5

[42] Schopfer P, Plachy C, Frahry G (2001) Release of reactive oxygen intermediates (superoxide radicals, hydrogen peroxide, and hydroxyl radicals) and peroxidase in germinating radish seeds controlled by light, gibberellin, and abscisic acid. Plant Physiol 125: 1591–1602. 11299341 10.1104/pp.125.4.1591 PMC88817

[43] Zhong S, Zhao M, Shi T, Shi H, An F, et al (2009) EIN3/EIL1 cooperate with PIF1 to prevent photo-oxidation and to promote greening of Arabidopsis seedlings. Proc Natl Acad Sci U S A 106: 21431–21436. 19948955 10.1073/pnas.0907670106 PMC2795496

[44] Zhou F, Andersen CH, Burhenne K, Fischer PH, Collinge DB, et al (2000) Proton extrusion is an essential signalling component in the HR of epidermal single cells in the barley-powdery mildew interaction. Plant J 23: 245–254. 10929118 10.1046/j.1365-313x.2000.00777.x

[45] Gadjev I, Vanderauwera S, Gechev TS, Laloi C, Minkov IN, et al (2006) Transcriptomic footprints disclose specificity of reactive oxygen species signaling in Arabidopsis. Plant Physiol 141: 436–445. 16603662 10.1104/pp.106.078717 PMC1475436

[46] Shi Y, Tian S, Hou L, Huang X, Zhang X, et al (2012) Ethylene signaling negatively regulates freezing tolerance by repressing expression of CBF and type-A ARR genes in Arabidopsis. Plant Cell 24: 2578–2595. 22706288 10.1105/tpc.112.098640 PMC3406918

[47] Chen H, Xue L, Chintamanani S, Germain H, Lin H, et al (2009) ETHYLENE INSENSITIVE3 and ETHYLENE INSENSITIVE3-LIKE1 repress SALICYLIC ACID INDUCTION DEFICIENT2 expression to negatively regulate plant innate immunity in Arabidopsis. Plant Cell 21: 2527–2540. 19717619 10.1105/tpc.108.065193 PMC2751940

[48] Lingam S, Mohrbacher J, Brumbarova T, Potuschak T, Fink-Straube C, et al (2011) Interaction between the bHLH transcription factor FIT and ETHYLENE INSENSITIVE3/ETHYLENE INSENSITIVE3-LIKE1 reveals molecular linkage between the regulation of iron acquisition and ethylene signaling in Arabidopsis. Plant Cell 23: 1815–1829. 21586684 10.1105/tpc.111.084715 PMC3123957

[49] Zhao Q, Guo HW (2011) Paradigms and paradox in the ethylene signaling pathway and interaction network. Mol Plant 4: 626–634. 21690206 10.1093/mp/ssr042

[50] Liu Z, Wu Y, Yang F, Zhang Y, Chen S, et al (2013) BIK1 interacts with PEPRs to mediate ethylene-induced immunity. Proc Natl Acad Sci U S A 110: 6205–6210. 23431184 10.1073/pnas.1215543110 PMC3625333

[51] Yanagisawa S, Yoo SD, Sheen J (2003) Differential regulation of EIN3 stability by glucose and ethylene signalling in plants. Nature 425: 521–525. 14523448 10.1038/nature01984

[52] Zhang L, Li Z, Quan R, Li G, Wang R, et al (2011) An AP2 domain-containing gene, ESE1, targeted by the ethylene signaling component EIN3 is important for the salt response in Arabidopsis. Plant Physiol 157: 854–865. 21832142 10.1104/pp.111.179028 PMC3192559

[53] Wu L, Zhang Z, Zhang H, Wang XC, Huang R (2008) Transcriptional modulation of ethylene response factor protein JERF3 in the oxidative stress response enhances tolerance of tobacco seedlings to salt, drought, and freezing. Plant Physiol 148: 1953–1963. 18945933 10.1104/pp.108.126813 PMC2593663

[54] Apel K, Hirt H (2004) Reactive oxygen species: metabolism, oxidative stress, and signal transduction. Annu Rev Plant Biol 55: 373–399. 15377225 10.1146/annurev.arplant.55.031903.141701

[55] Roxas VP, Lodhi SA, Garrett DK, Mahan JR, Allen RD (2000) Stress tolerance in transgenic tobacco seedlings that overexpress glutathione S-transferase/glutathione peroxidase. Plant Cell Physiol 41: 1229–1234. 11092907 10.1093/pcp/pcd051

[56] Tseng MJ, Liu CW, Yiu JC (2007) Enhanced tolerance to sulfur dioxide and salt stress of transgenic Chinese cabbage plants expressing both superoxide dismutase and catalase in chloroplasts. Plant Physiol Biochem 45: 822–833. 17851086 10.1016/j.plaphy.2007.07.011

[57] Badawi GH, Kawano N, Yamauchi Y, Shimada E, Sasaki R, et al (2004) Over-expression of ascorbate peroxidase in tobacco chloroplasts enhances the tolerance to salt stress and water deficit. Physiol Plant 121: 231–238. 15153190 10.1111/j.0031-9317.2004.00308.x

[58] De Boer K, Tilleman S, Pauwels L, Vanden Bossche R, De Sutter V, et al (2011) APETALA2/ETHYLENE RESPONSE FACTOR and basic helix-loop-helix tobacco transcription factors cooperatively mediate jasmonate-elicited nicotine biosynthesis. Plant J 66: 1053–1065. 21418355 10.1111/j.1365-313X.2011.04566.x

[59] Shyu C, Figueroa P, Depew CL, Cooke TF, Sheard LB, et al (2012) JAZ8 lacks a canonical degron and has an EAR motif that mediates transcriptional repression of jasmonate responses in Arabidopsis. Plant Cell 24: 536–550. 22327740 10.1105/tpc.111.093005 PMC3315231

[60] Li Z, Peng J, Wen X, Guo H (2013) Ethylene-insensitive3 is a senescence-associated gene that accelerates age-dependent leaf senescence by directly repressing miR164 transcription in Arabidopsis. Plant Cell 25: 3311–3328. 24064769 10.1105/tpc.113.113340 PMC3809534

[61] Jefferson RA, Kavanagh TA, Bevan MW (1987) GUS fusions: beta-glucuronidase as a sensitive and versatile gene fusion marker in higher plants. EMBO J 6: 3901–3907. 3327686 10.1002/j.1460-2075.1987.tb02730.x PMC553867

[62] Peng JY, Li ZH, Xiang H, Huang JH, Jia SH, et al (2005) Preliminary studies on differential defense responses induced during plant communication. Cell Res 15: 187–192. 15780181 10.1038/sj.cr.7290285

[63] Clough SJ, Bent AF (1998) Floral dip: a simplified method for Agrobacterium-mediated transformation of Arabidopsis thaliana. Plant J 16: 735–743. 10069079 10.1046/j.1365-313x.1998.00343.x

