# Supplementary figures and images for: Salt-Induced Stabilization of EIN3/EIL1 Confers Salinity Tolerance by Deterring ROS Accumulation in Arabidopsis
Source: PLoS Genet. 2014 Oct 16;10(10):e1004664. doi: 10.1371/journal.pgen.1004664 (PMC4199496; doi:10.1371/journal.pgen.1004664)

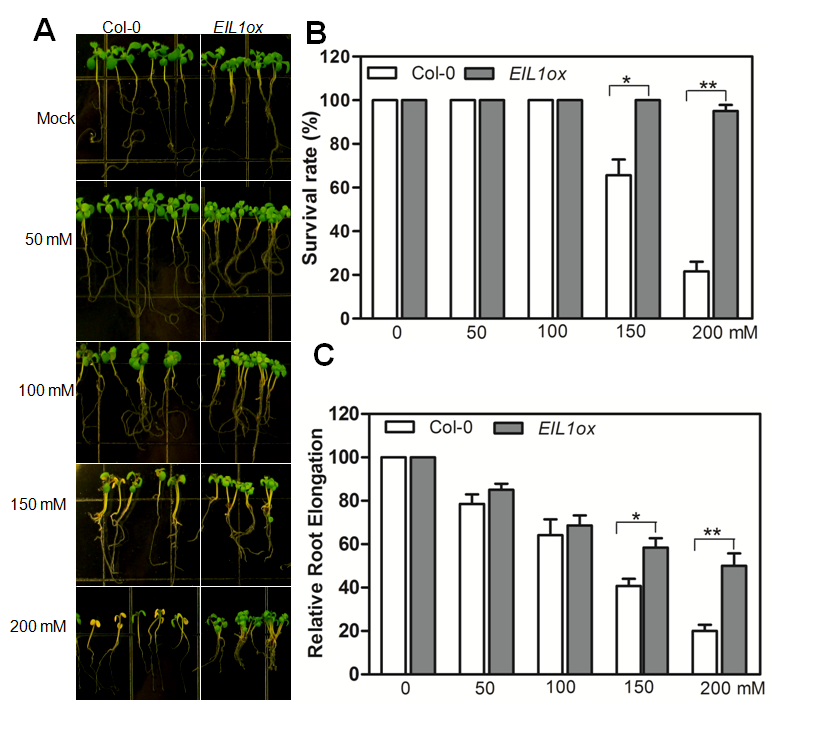

Supplement: Figure S1 — Overexpression of EIL1 increases salt tolerance. (A) Seedlings were grown on MS medium for 5 d and then transferred onto MS medium with 0, 50, 100, 150, 200 mM NaCl for 7 d. (B) Survival rate and (C) relative root elongation of seedlings shown in (A). Seedling death was scored as complete bleaching of cotyledons and leaves. Root length of seedlings transferred to MS medium without salt was set to 100%. Values are mean ± SD from 30 seedlings per replicate (n = 3 replicates). (Student's t test, *P<0.05 and **P<0.01). (TIF) [file pgen.1004664.s001.tif]

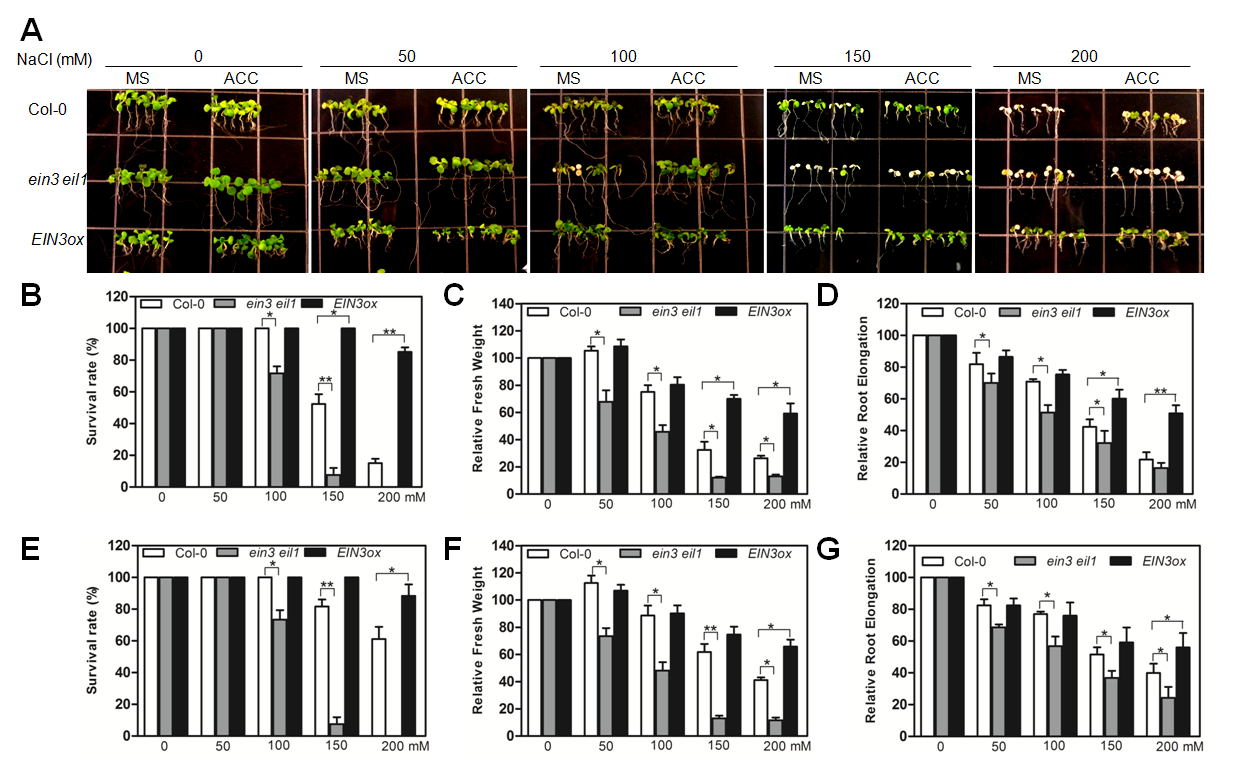

Supplement: Figure S2 — ACC pretreatment or enhanced ethylene signaling increases salt tolerance. (A) Seedlings were grown on MS medium with or without 10 µM ACC for 5 d and then transferred onto MS medium with 0, 50, 100, 150, 200 mM NaCl for 7 d. (B) and (E) Survival rate of seedlings shown in (A). Seedlings grown on MS (B) or ACC (E) were transferred to MS medium supplemented with NaCl. Seedling death was scored as complete bleaching of cotyledons and leaves. Values are mean ± SD from 30 seedlings per replicate (n = 3 replicates). (Student's t test, *P<0.05 and **P<0.01). (C) and (F) Relative fresh weight of seedlings shown in (A). Seedlings grown on MS (C) or ACC (F) were transferred to MS medium supplemented with NaCl. Fresh weight of seedlings transferred to MS medium without salt was set to 100%. Values are mean ± SD from 30 seedlings per replicate (n = 3 replicates). (Student's t test, *P<0.05 and **P<0.01). (D) and (G) Relative root elongation of seedlings shown in (A). Seedlings grown on MS (D) or ACC (G) were transferred to MS medium supplemented with NaCl. Root length of seedlings transferred to MS medium without salt was set to 100%. Values are mean ± SD from 30 seedlings per replicate (n = 3 replicates). (Student's t test, *P<0.05 and **P<0.01). (TIF) [file pgen.1004664.s002.tif]

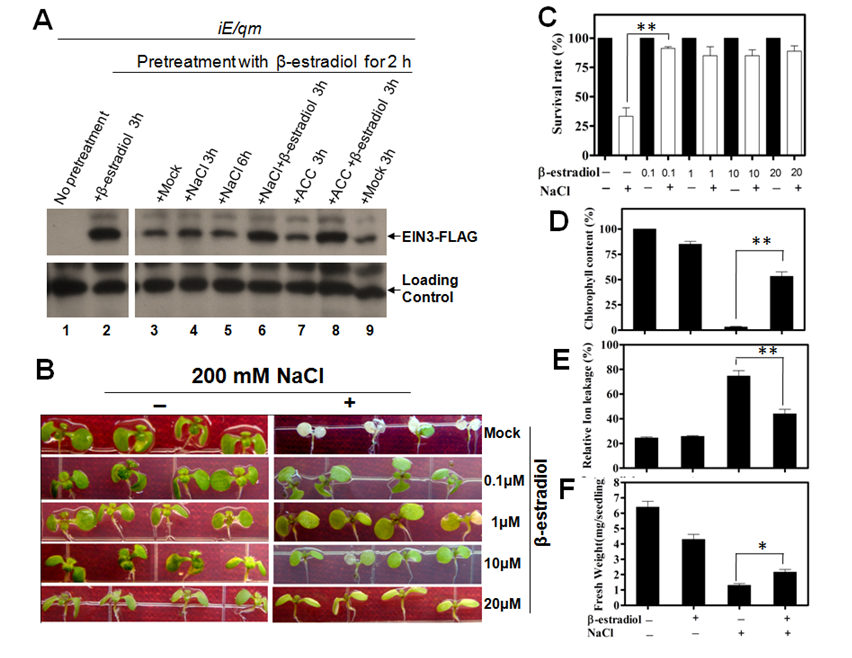

Supplement: Figure S3 — Inducible overexpression of EIN3 is sufficient to confer salt tolerance. (A) Western blot analysis of EIN3-FLAG protein accumulation. β-estradiol-inducible EIN3-FLAG in the ein3 eil1 ebf1 ebf2 background (iE/qm) seedlings grown on MS for 5 d and pretreated with 100 µM β-estradiol for 2 h. After washing with water for 3 times, the seedlings were then treated with 100 µM ACC or 200 mM NaCl for another 3 h or 6 h. Proteins were extracted and subjected to immunoblots using anti-FLAG antibody. Experiments were repeated three times with similar results. (B) Plants were grown on MS medium for 5 d and then transferred onto MS medium supplemented with (+) or without (−) 200 mM NaCl in the presence of indicated concentrations of estradiol for 3 d. (C) Survival rate of plants shown in (B). Values are mean ± SD from 30 seedlings per replicate (n = 3 replicates). (Student's t test, *P<0.05 and **P<0.01). (D) to (F) Quantification of total chlorophyll content (D), relative electrolyte leakage (E) and fresh weights (F) of iE/qm seedlings grown on MS medium supplemented with or without 200 mM NaCl and 1 µM β-estradiol for 3 d. Values are mean ± SD from 25 seedlings per replicate (n = 5 replicates). (Student's t test, *P<0.05 and **P<0.01). (TIF) [file pgen.1004664.s003.tif]

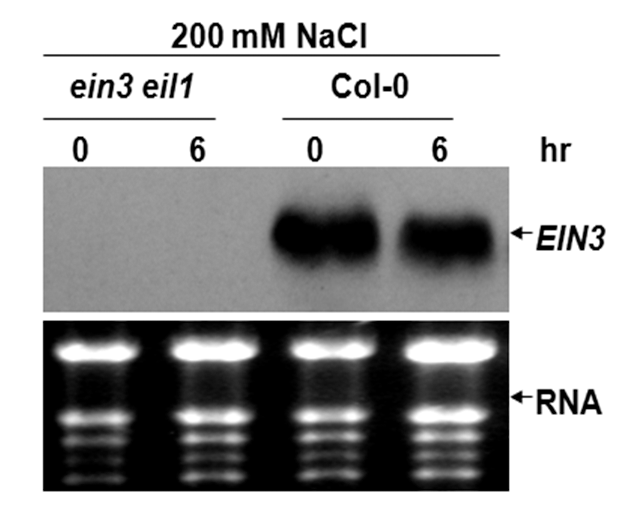

Supplement: Figure S4 — Salt treatment did not alter EIN3 mRNA level. 5-d-old seedlings grown on MS medium were transferred into liquid culture medium with or without 200 mM NaCl for 6 h. Total RNA was extracted and subjected to northern blots analysis with labeled EIN3 cDNA probes. Ethidium bromide staining of the gel was shown in the bottom image as loading controls. (TIF) [file pgen.1004664.s004.tif]

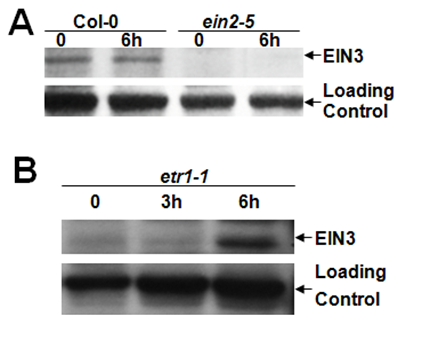

Supplement: Figure S5 — Western blot analyses of EIN3 protein accumulation in mock-treated Col-0 and ein2-5 for 6 h, and in salt-treated etr1-1 for 3 h and 6 h. (A) EIN3 protein accumulation in mock-treated Col-0 and ein2-5. 5-d-old Col-0 or ein2-5 seedlings grown on MS medium were transferred into liquid culture medium for 6 h. Total protein was extracted and subjected to Western blot analysis with anti-EIN3 antibody. A nonspecific band was used as a loading control. Experiments were repeated three times with similar results. (B) EIN3 protein accumulation in salt-treated etr1-1. 5-d-old etr1-1 seedlings were treated with 200 mM NaCl for 3 h and 6 h. Protein was extracted and subjected to immunoblots using anti-EIN3 antibody. A nonspecific band was used as a loading control. (TIF) [file pgen.1004664.s005.tif]

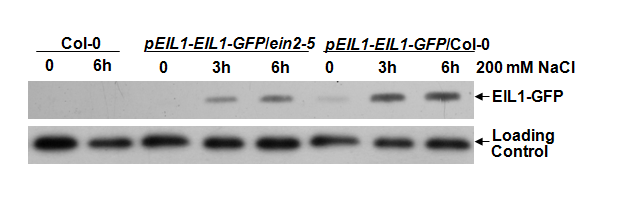

Supplement: Figure S6 — Salt treatment promotes protein accumulation of EIL1 in both EIN2-dependent and EIN2-independent manners. 5-d-old seedlings of pEIL1-EIL1-GFP/ein2-5 and pEIL1-EIL1-GFP/Col-0 were treated with 200 mM NaCl for 3 h and 6 h. 5-d-old Col-0 seedlings treated with 200 mM NaCl for 6 h were used as the negative control. Protein was extracted and subjected to immunoblots using anti-GFP antibody. A nonspecific band was used as a loading control. (TIF) [file pgen.1004664.s006.tif]

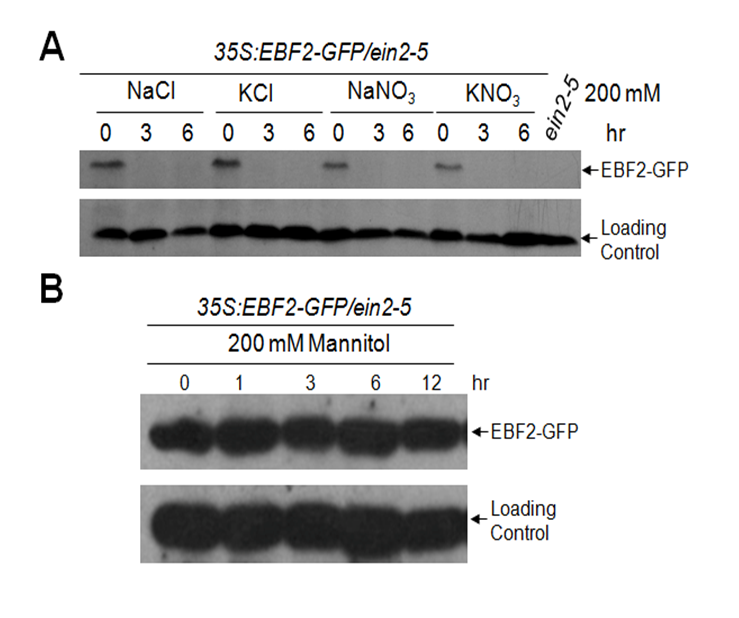

Supplement: Figure S7 — Ionic stresses but not osmotic stress regulate EBF2-GFP protein stability in an EIN2 independent manner. (A) Immunoblot assay of EBF2-GFP protein in ein2-5 background upon treatment with NaCl, KCl, NaNO3 and KNO3. Transgenic seedlings grown on MS medium for 5 d were subjected to 200 mM NaCl, 200 mM KCl, 200 mM NaNO3 or 200 mM KNO3 for 3 h and 6 h. Protein levels of EBF2-GFP were analyzed by Western blot using an anti-GFP antibody. Experiments were repeated three times with similar results. (B) Immunoblot assay of EBF2-GFP protein in ein2-5 background upon treatment with mannitol. Transgenic seedlings grown on medium for 5 d were subjected to 200 mM mannitol for indicated time. Protein levels of EBF2-GFP were analyzed by Western blot using an anti-GFP antibody. Experiments were repeated three times with similar results. (TIF) [file pgen.1004664.s007.tif]

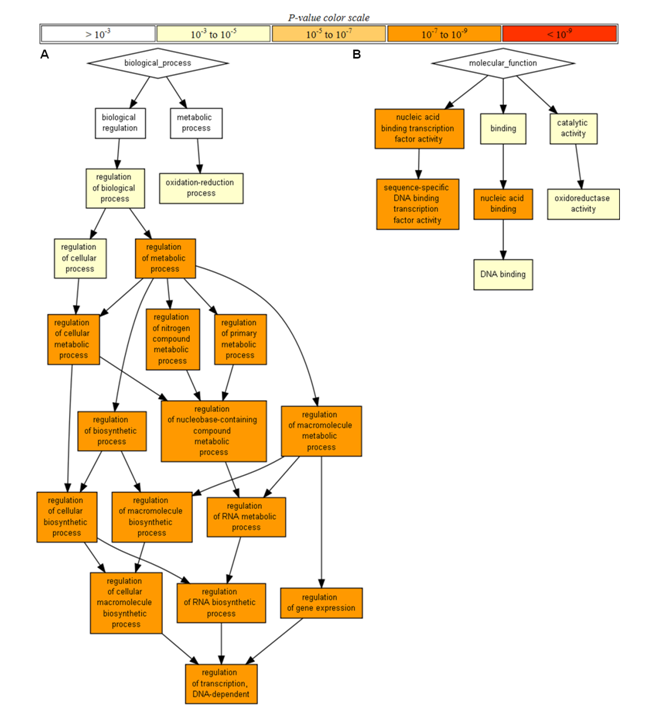

Supplement: Figure S8 — The enriched GO terms in SIED genes. (A and B) The network graphs show Gorilla visualization of GO terms for SIED genes: biological process (A) and molecular function (B). Colored nodes represent GO terms that are significantly overrepresented (P value<0.05), with the shade indicating significance as shown in the color bar. A more detailed analysis of the GO categories is shown in Table S1 online. (TIF) [file pgen.1004664.s008.tif]

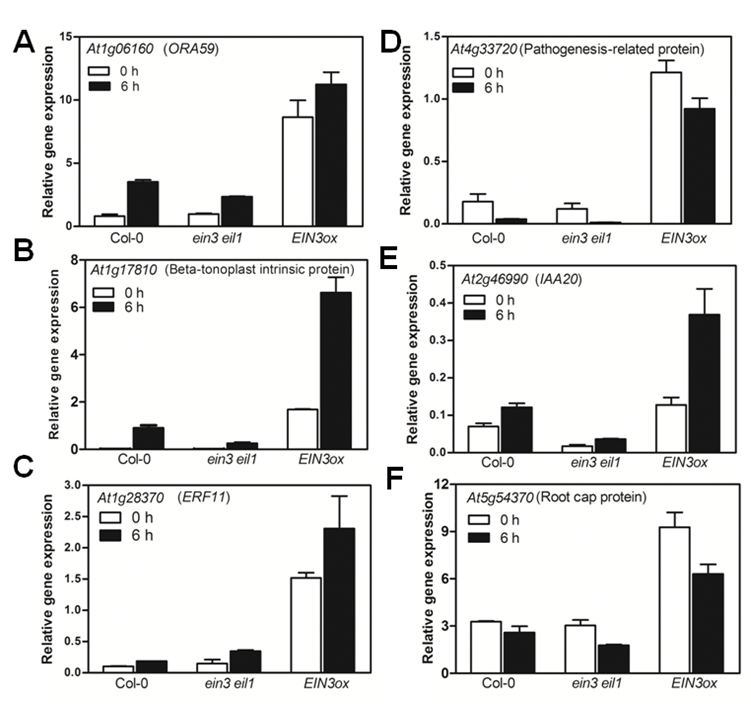

Supplement: Figure S9 — qRT-PCR analysis of selected EIN3-induced genes. (A–F) qRT-PCR analysis of selected EIN3-induced genes in Col-0, ein3eil1 and EIN3ox seedlings. 5-d-old seedlings grown on MS medium were transferred into liquid culture medium with or without 200 mM NaCl for 6 h. Total RNA was extracted and subjected to qRT-PCR analysis. Three biological replicates and two technical replicates were performed. (TIF) [file pgen.1004664.s009.tif]

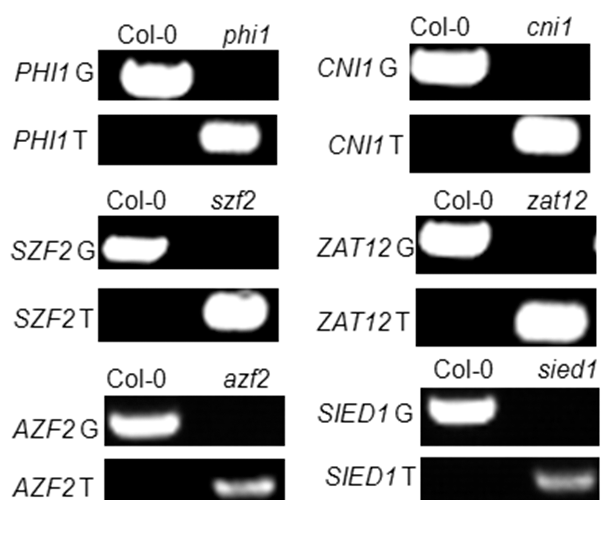

Supplement: Figure S10 — PCR genotyping of the Salk T-DNA mutants of the six SIED genes. 5-d-old seedlings were subjected to DNA extraction and subsequent PCR analysis. G, gene specific primers; T, the T-DNA left border primer used in combination with gene specific primers. (TIF) [file pgen.1004664.s010.tif]

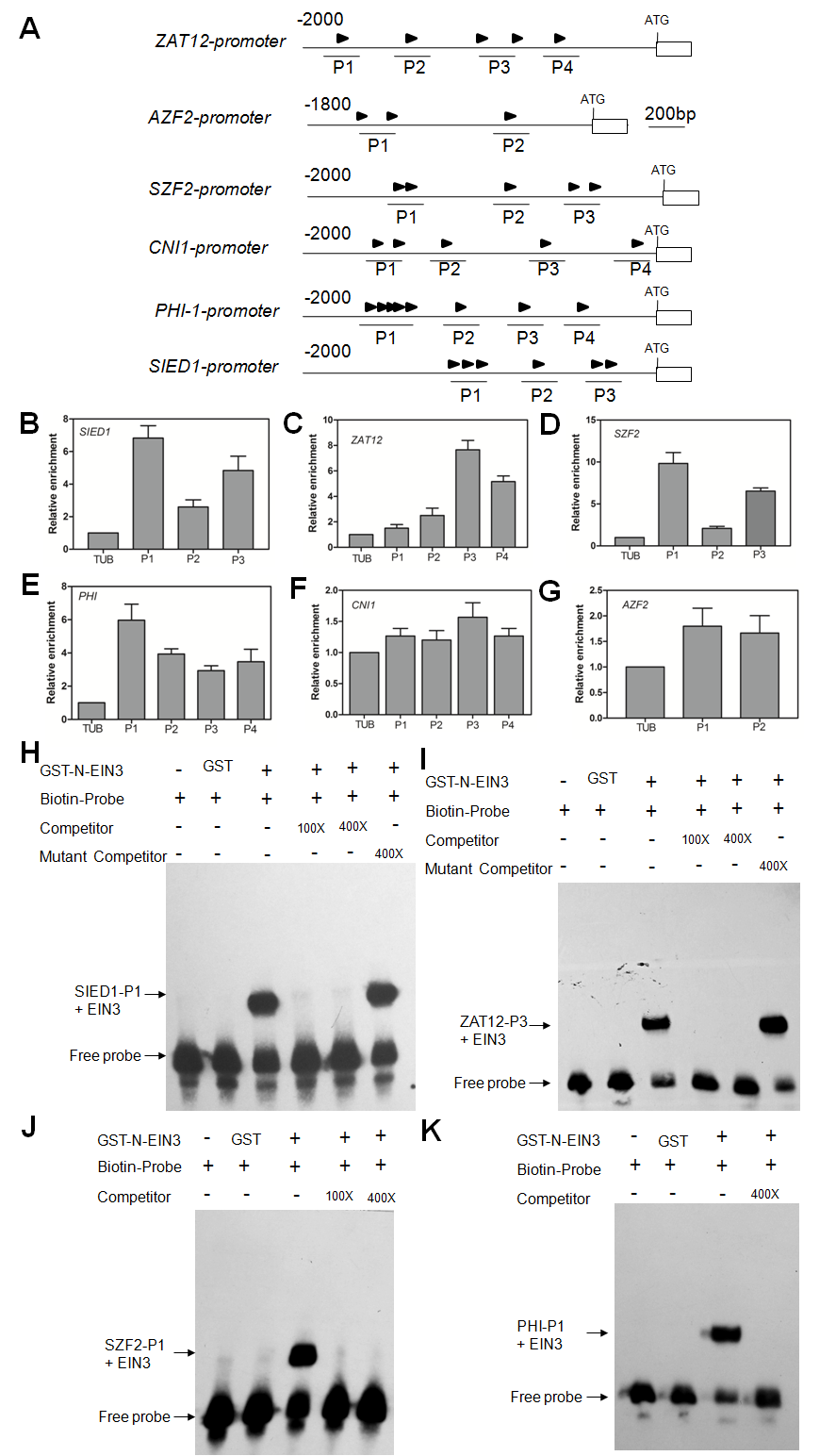

Supplement: Figure S11 — EIN3 protein directly binds to the promoters of several SIED genes. (A) Schematic diagrams of putative EIN3 Binding Site (EBS) (arrows) in the promoters of six SIED genes and DNA fragments (P1, P2, P3 and P4) used for ChIP or EMSA experiments. The 1.8 or 2.0 kb upstream sequences are shown, and the translational start site (ATG) is shown at position +1. (B)–(G) Chromatin immunoprecipitation (ChIP)-qPCR assays of the promoter regions of SIED genes from DNA of Col-0 seedlings with anti-EIN3 antibody. A Tubulin 8 fragment was amplified as control. Three biological replicates and two technique replicates were performed with similar results. (H)–(K) EMSA showing the interaction between the EBS containing region of SIED genes and EIN3 protein. GST-tagged EIN3 N-terminus (aa 141–352) fusion protein was incubated with biotin-labeled DNA fragment. Competition for the biotin-labeled promoter region was done by adding an excess of unlabeled wild-type probe (competitor) or mutated probe (mutant competitor). Two biological replicates and two technique replicates were performed with similar results. (TIF) [file pgen.1004664.s011.tif]

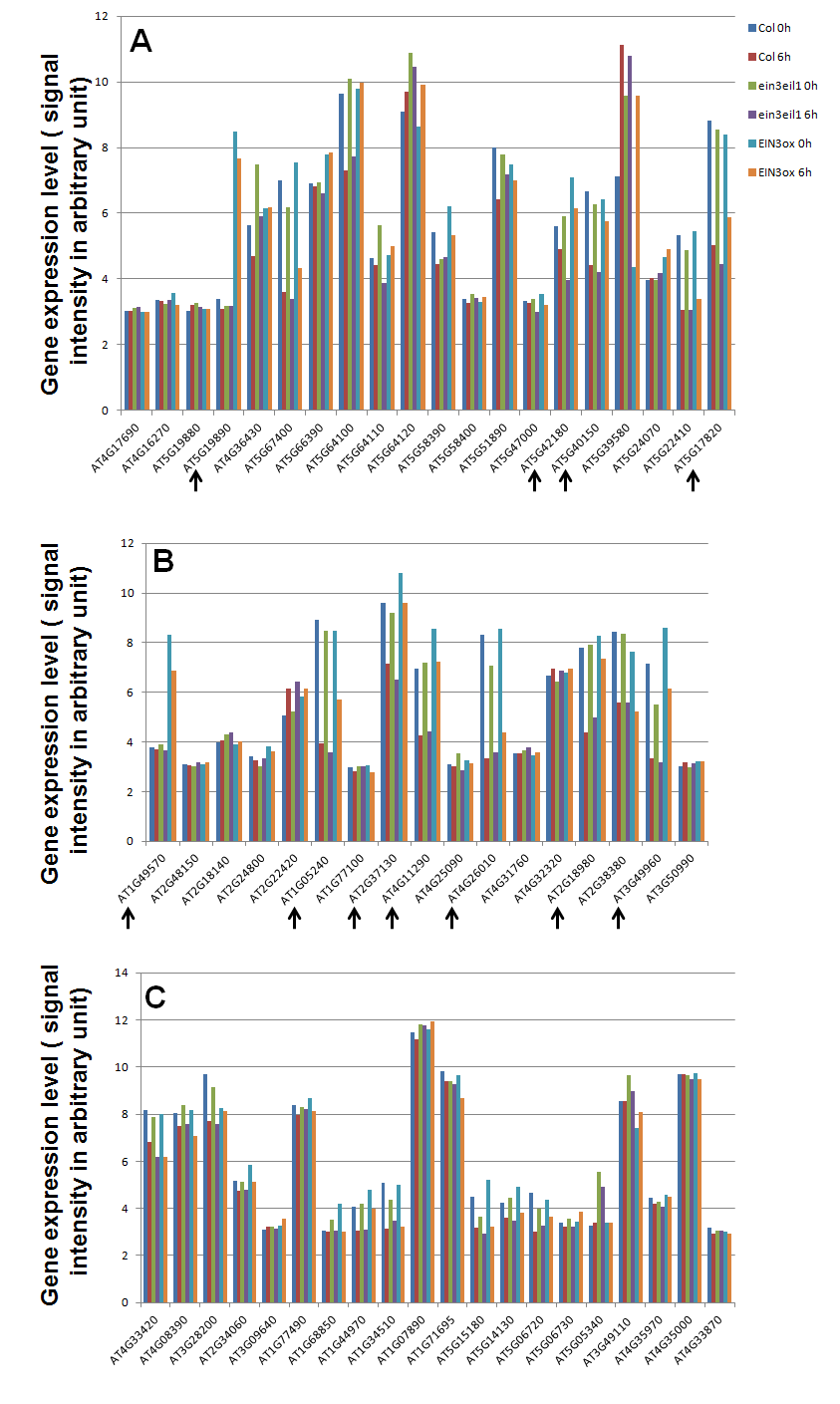

Supplement: Figure S12 — Transcriptome profiling of genes encoding PODs in Col-0, ein3 eil1 and EIN3ox (A–C). Transcriptome profiling and data analysis were performed as described in “Methods”. Genes Exhibiting higher expression level in EIN3ox plants were highlighted with arrows. (TIF) [file pgen.1004664.s012.tif]

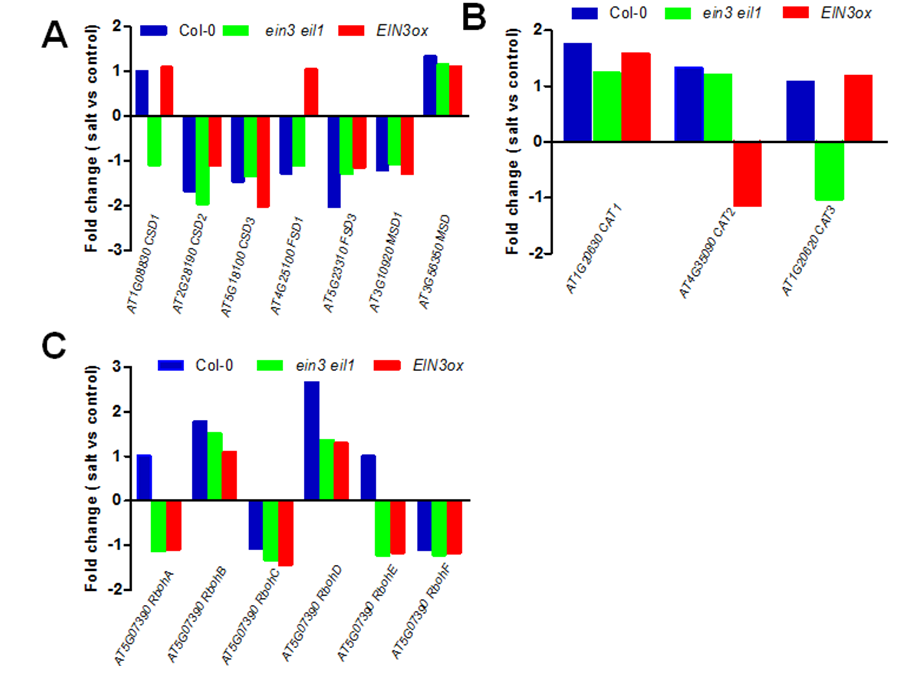

Supplement: Figure S13 — Transcriptome profiling of genes encoding Superoxide Dismutase (SOD) (A), Catalase (CAT1-3) (B) and NADPH Oxidase (RobhA-F) (C) in Col-0, ein3 eil1 and EIN3ox. (TIF) [file pgen.1004664.s013.tif]

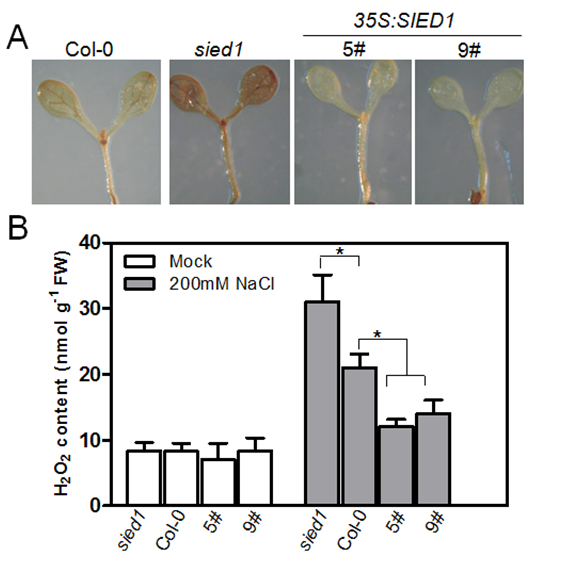

Supplement: Figure S14 — ROS accumulation in salt-treated Col-0, sied1 and 35S:SIED1 plants. (A) DAB staining of seedlings under normal conditions or salt treatment. Seedlings grown on MS medium for 5 d were treated with or without 200 mM NaCl for 6 h, and used for DAB staining. Experiments were repeated three times with similar results. (B) H2O2 content in the seedlings in (A) (Student's t test, *P<0.05 and **P<0.01). (TIF) [file pgen.1004664.s014.tif]

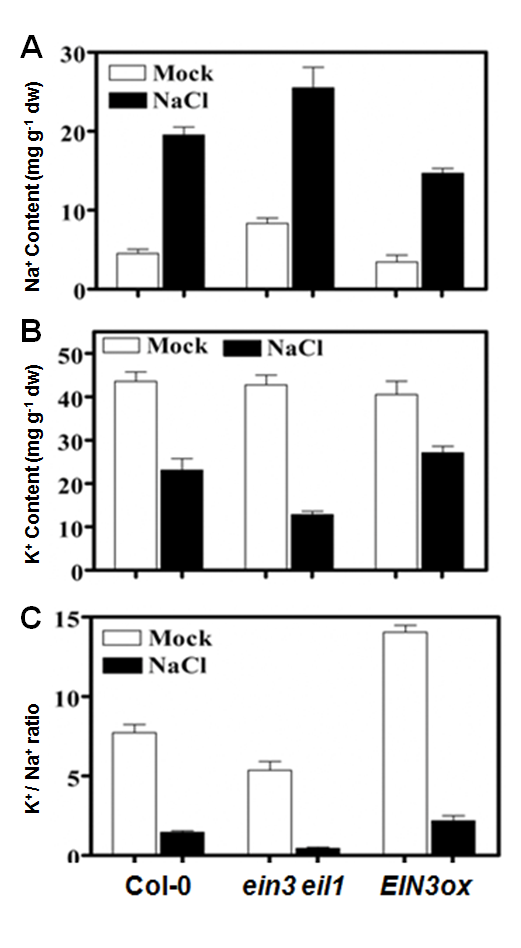

Supplement: Figure S15 — Na+ and K+ Content in Salt-treated Col-0, ein3eil1 and EIN3ox plants. 5-d-old seedlings grown on MS medium were transferred into liquid culture medium with or without 200 mM NaCl for 6 h. Na+ content (A), K+ content (B) and K+ / Na+ ration (C) in the whole seedlings were examined. Values are mean ± SD (n = 3 replicates). dw, dry weight. (TIF) [file pgen.1004664.s015.tif]
