# Supplementary material for: Salt-Induced Stabilization of EIN3/EIL1 Confers Salinity Tolerance by Deterring ROS Accumulation in Arabidopsis
Source: PLoS Genet. 2014 Oct 16;10(10):e1004664. doi: 10.1371/journal.pgen.1004664 (PMC4199496; doi:10.1371/journal.pgen.1004664)
Supplement: Table S1 — Salt-Induced EIN3/EIL1-Dependent (SIED) genes (114). Genes highlighted in red are direct targets of EIN3 that were identified by ChIP-Seq experiments. (DOC) [file pgen.1004664.s016.doc]

**Table S1.** Salt-Induced EIN3/EIL1-Dependent (SIED) Genes (114). Genes highlighted in red are the direct targets of EIN3 that were identified by ChIP-Seq experiments.

| Locus | Col-0 | *ein3eil1* | *EIN3ox* | Description |
| --- | --- | --- | --- | --- |
| **Transcription (31)** | |  |  |  |
| ERF/AP2 family (11) | | | | |
| *AT3G23240* | 25.39 | 7.93 | 32.69 | ERF1 |
| *AT5G47220* | 31.99 | 12.2 | 44.73 | ERF2 |
| *AT5G47230* | 7.77 | 6.49 | 10.31 | ERF5 |
| *AT4G17490* | 13.52 | 6.93 | 36.86 | ERF6 |
| *AT2G44840* | 52.05 | 23.27 | 65.23 | ERF13 |
| *AT5G61600* | 8.83 | 5.22 | 9.26 | ERF104 |
| *AT1G06160* | 6.16 | 1.64 | 10.88 | ORA59 |
| *AT1G64380* | 5.39 | 4.13 | 5.45 | Encodes a member of the ERF/AP2 family |
| *AT5G51190* | 6.36 | 4.43 | 7.61 | Encodes a member of the ERF/AP2 family |
| *AT5G61890* | 7.53 | 3.46 | 9.40 | Encodes a member of the ERF/AP2 family |
| *AT3G11580* | 5.04 | 3.31 | 7.13 | AP2/B3-like transcriptional factor family protein |
| ZIM family (4) | | | | |
| *AT1G74950* | 17.77 | 7.57 | 21.92 | JAZ2 |
| *AT3G17860* | 5.69 | 4.76 | 9.19 | JAZ3 |
| *AT2G34600* | 176.3 | 27.49 | 197.55 | JAZ7 |
| *AT1G30135* | 39.26 | 16.44 | 63.04 | JAZ8 |
| Zinc finger protein (9) | | | | |
| *AT2G40140* | 8.22 | 5.98 | 9.67 | SALT-INDUCIBLE ZINC FINGER 2 |
| *AT1G51700* | 8.23 | 3.93 | 8.86 | Dof zinc finger protein 1 |
| *AT2G37430* | 24.15 | 12.46 | 37.92 | Putative C2H2-type zinc finger protein |
| *AT1G76410* | 5.69 | 3.49 | 13.53 | Putative RING zinc finger protein, ATL8 |
| *AT2G19810* | 9.52 | 5.66 | 12.38 | OXIDATION-RELATED ZINC FINGER 1 |
| *AT3G19580* | 20.26 | 8.89 | 25.59 | Cys2/His2-type zinc finger protein(AZF2) |
| *AT3G55980* | 10.75 | 5.23 | 25.63 | Putative protein zinc finger transcription factor (PEI1) |
| *AT5G59820* | 32.09 | 9.53 | 35.43 | Zinc finger protein Zat12 |
| *AT5G27420* | 19.98 | 4.82 | 28.37 | RING-H2 zinc finger protein ATL6/CNI1 |
| Others (8) |  |  |  |  |
| *AT1G10170* | 7.33 | 4.66 | 9.70 | Protein similar to transcriptional repressor NF-X1 |
| *AT1G22810* | 31.89 | 21.63 | 75.95 | TINY-like transcription factor |
| *AT2G22850* | 15.96 | 8.33 | 20.69 | Putative embryo-abundant protein |
| *AT1G42990* | 6.10 | 4.31 | 8.08 | bZIP transcription factor,bZIP60 |
| *AT2G26150* | 12.68 | 7.67 | 13.60 | Heat shock transcription factor HSFA2 |
| *AT3G46600* | 12.41 | 6.31 | 12.89 | GRAS family transcription factor |
| *AT3G56400* | 5.24 | 2.56 | 5.25 | WRKY4 |
| *AT4G01250* | 6.92 | 5.89 | 28.49 | WRKY22 |
| **Oxidoreductase activity (8)** | | |  |  |
| *AT1G06620* | 16.87 | 8.27 | 45.35 | Oxidoreductase |
| *AT1G28480* | 57.32 | 16.2 | 66.46 | Glutaredoxin |
| *AT1G30700* | 14.26 | 2.49 | 17.56 | Putative reticuline oxidase-like protein |
| *AT3G09940* | 14.09 | 4.9 | 15.09 | Putative monodehydroascorbate reductase (NADH) |
| *AT4G37370* | 37.35 | 9.17 | 224.94 | Cytochrome P450 |
| *AT5G05600* | 64.51 | 7.37 | 85.4 | Leucoanthocyanidin dioxygenase-like protein |
| *AT5G39580* | 15.88 | 2.34 | 36.95 | Peroxidase ATP24a |
| *AT5G63450* | 18.21 | 11.12 | 19.57 | Cytochrome P450-like protein |
| **Electron transport or energy pathways (4)** | | | | |
| *AT1G30720* | 15.06 | 1.85 | 19.66 | Putative reticuline oxidase-like protein |
| *AT2G41730* | 109.9 | 9.18 | 227.95 | Putative protein in anaerobic respiration |
| *AT3G09350* | 15.8 | 8.38 | 15.93 | FES1A |
| *AT5G20230* | 14.86 | 2.64 | 23.77 | Blue copper binding protein |
| **Defense response (18)** | | | | |
| *AT1G02920* | 10.02 | 1.17 | 10.51 | Glutathione S-transferase |
| *AT1G07400* | 49.84 | 19.1 | 52.84 | Heat shock protein |
| *AT1G15010* | 19.02 | 11.02 | 30.14 | Defense response to fungus |
| *AT1G19610* | 9.54 | 2 | 32.96 | Predicted to encode a PR (pathogenesis-related) protein |
| *AT1G35140* | 8.8 | 2.29 | 20.97 | Phosphate-induced (phi-1) |
| *AT1G54050* | 15.28 | 9.26 | 35.02 | Heat-shock protein |
| *AT1G72060* | 8.37 | 2.34 | 38.29 | Serine-type endopeptidase inhibitors |
| *AT2G26380* | 22.45 | 9.2 | 28.09 | Putative disease resistance protein |
| *AT2G38870* | 12.94 | 1.6 | 18.68 | Putative protease inhibitor |
| *AT2G39200* | 40.02 | 2.57 | 16.68 | MILDEW RESISTANCE LOCUS O 12 |
| *AT3G50980* | 8.57 | 4.26 | 8.29 | Dehydrin xero 1 (XERO1) |
| *AT4G12720* | 8.55 | 3.98 | 8.78 | NUDIX HYDROLASE HOMOLOG 7 |
| *AT4G17615* | 10.26 | 7.16 | 8.28 | CALCINEURIN B-LIKE PROTEIN(ATCBL1) |
| *AT4G21830* | 24.58 | 4.13 | 35.04 | Methionine sulfoxide reductase B7 |
| *AT2G43620* | 5.55 | -1.18 | 33.66 | Putative endochitinase |
| *AT5G15960* | 5.97 | 5.30 | 14.35 | Cold and ABA inducible protein KIN1 |
| *AT5G44910* | 6.92 | 2.32 | 7.40 | Toll-Interleukin-Resistance (TIR) domain family protein |
| *AT5G62490* | 11.81 | 7.46 | 14.95 | Protein expression is ABA- and stress-inducible |
| **Biosynthetic process and metabolism (5)** | | | | |
| *AT1G21110* | 13.33 | 2.95 | 19.46 | O-methyltransferase |
| *AT1G67980* | 33.13 | 6.57 | 42.75 | SAM : trans-caffeoyl-Coenzyme A 3-O-methyltransferase |
| *AT2G03760* | 47.85 | 7.93 | 61.18 | Putative steroid sulfotransferase |
| *AT3G54950* | 5.12 | 3.62 | 5.53 | Patatin-related phospholipases |
| *AT5G01880* | 5.47 | 5.29 | 6.55 | RING/U-box E3 ubiquitin ligase |
| **Transport (3)** | | | | |
| *AT1G08920* | 12.37 | 6.31 | 13.13 | Encodes ESL1, a transporter for monosaccharides. |
| *AT4G21680* | 7.7 | 4.98 | 15.14 | Peptide transporter - like protein peptide transporter (ptr1) |
| *AT5G40780* | 5.89 | 1.66 | 6.30 | Amino acid permease |
| **Protein kinase activity (6)** | | | | |
| *AT1G73500* | 13.26 | 7.7 | 27.63 | MKK9 |
| *AT2G30040* | 26.82 | 9.95 | 38.28 | MAPKKK14 |
| *AT3G53810* | 5.33 | 4.42 | 6.10 | Serine/threonine-specific kinase |
| *AT3G59480* | 7.32 | 4.54 | 11.88 | Fructokinase-like protein |
| *AT3G62260* | 7.37 | 4.47 | 7.83 | Putative protein phosphoprotein phosphatase |
| *AT5G55090* | 6.93 | 6.78 | 17.54 | MAPKKK15 |
| **Response to hormone (2)** | | | | |
| *AT4G26200* | 11.06 | 2.45 | 28.4 | ACC synthase7 (ACS7) |
| *AT5G13200* | 7.82 | 4.99 | 10.38 | ABA-responsive protein |
| **Calcium ion binding (2)** | | | | |
| *AT2G41100* | 16.16 | 3.82 | 17.91 | Calmodulin-like protein (TOUCH3) |
| *AT5G54490* | 24.75 | 8.83 | 28.38 | PINOID-BINDING PROTEIN 1 (PBP1) |
| **Cell organization and biogenesis (1)** | | | | |
| *AT5G40260* | 33 | 9.23 | 40.31 | SWEET8 |
| **Unknown (33)** | | | | |
| *AT1G05340* | 11.48 | 5.29 | 11.21 | Unknown protein |
| *AT1G12950* | 5.95 | 5.61 | 9.97 | Unknown protein |
| *AT1G21010* | 12 | 5.23 | 18.99 | Unknown protein |
| *AT1G21525* | 7.43 | 3.78 | 10.89 | Hypothetical protein |
| *AT1G28380* | 5.93 | 3.28 | 6.22 | Unknown protein |
| *AT1G52200* | 5.57 | 1.71 | 9.01 | Unknown protein |
| *AT1G65500* | 6.41 | -1.26 | 6.81 | Unknown protein |
| *AT1G23710* | 22.26 | 6.8 | 23.7 | Unknown protein |
| *AT1G25400* | 20.13 | 7.3 | 28.89 | Unknown protein |
| *AT1G53885* | 36 | 12.82 | 46.56 | Expressed protein |
| *AT1G65510* | 5.81 | 1.73 | 23.9 | Hypothetical protein |
| *AT1G69890* | 17.53 | 11.73 | 23.8 | Hypothetical protein |
| *AT1G76600* | 32.48 | 8.44 | 54.34 | Unknown protein |
| *AT2G18210* | 33.51 | 8.5 | 47.15 | Unknown protein |
| *AT2G18680* | 13.47 | 8.13 | 26.49 | Unknown protein |
| *AT2G23170* | 7.33 | 5 | 38.38 | Unknown protein |
| *AT2G25735* | 26.17 | 6.4 | 32.73 | Expressed protein |
| *AT2G32190* | 11.6 | 4.94 | 15.09 | Unknown protein |
| *AT3G25655* | 13.3 | 6.73 | 24.94 | Expressed protein |
| *AT3G18690* | 5.03 | 3.83 | 8.65 | Hypothetical protein |
| *AT3G22160* | 6.76 | 3.66 | 13.44 | VQ motif-containing protein |
| *AT3G48510* | 7.51 | 6.01 | 8.11 | Hypothetical protein |
| *AT3G51660* | 5.74 | 1.45 | 7.78 | Unknown protein |
| *AT4G20000* | 6.02 | 2.38 | 7.49 | Hypothetical protein |
| *AT4G28703* | 22.95 | 10.74 | 26.83 | Expressed protein |
| *AT4G29285* | 17.52 | 8.98 | 20.91 | Expressed protein |
| *AT4G36500* | 13.8 | 4.77 | 26.27 | Putative protein |
| *AT5G22270* | 17.51 | 4.88 | 16.92 | Putative protein |
| *AT5G64900* | 5.33 | 2.98 | 32.51 | Unknown protein |
| *AT5G12340* | 79.13 | 20.19 | 93.48 | Putative protein |
| *AT5G55620* | 24.75 | 8.83 | 28.38 | Putative protein |
| *AT5G05850* | 6.53 | 4.56 | 9.09 | Putative protein |
| *AT5G52750* | 7.13 | 2.66 | 8.71 | Unknown protein |
