# Supplementary material for: Salt-Induced Stabilization of EIN3/EIL1 Confers Salinity Tolerance by Deterring ROS Accumulation in Arabidopsis
Source: PLoS Genet. 2014 Oct 16;10(10):e1004664. doi: 10.1371/journal.pgen.1004664 (PMC4199496; doi:10.1371/journal.pgen.1004664)
Supplement: Table S2 — Salt-Repressed EIN3/EIL1-Dependent (SRED) genes (14). (DOC) [file pgen.1004664.s017.doc]

**Table S2**. Salt-Repressed EIN3/EIL1-Dependent (SRED) Genes (14).

| Locus | Col-0 | *ein3eil1* | *EIN3ox* | Description |
| --- | --- | --- | --- | --- |
| **Transport** |  |  |  |  |
| *AT1G32450* | -8.30 | -7.10 | -13.34 | Nitrate transporter 1.5 |
| *AT2G25810* | -8.96 | -6.05 | -15.91 | Tonoplast intrinsic protein 4;1 |
| *AT2G48140* | -6.23 | -4.80 | -12.78 | Embryo sac development arrest 4 |
| *AT3G53980* | -5.10 | -1.92 | -6.29 | Bifunctional inhibitor/lipid-transfer |
| **Metabolic process** | |  |  |  |
| *AT3G62040* | -5.80 | -5.17 | -8.81 | Haloacid dehalogenase-like hydrolase protein |
| *AT5G66280* | -5.17 | -3.30 | -5.25 | GDP-D-mannose 4,6-dehydratase |
| *AT5G57530* | -6.31 | -2.40 | -9.96 | xyloglucan endotransglucosylase/hydrolase 12 |
| **Defense response** | |  |  |  |
| *AT5G63660* | -7.01 | -3.29 | -7.59 |  |
| **Cell wall organization** | |  |  |  |
| *AT5G04960* | -7.50 | -5.15 | -9.01 | Plant invertase/pectin methylesterase inhibitor s |
| *AT5G35190* | -10.28 | -5.32 | -14.84 | Proline-rich extensin-like protein |
| **Others** |  |  |  |  |
| *AT1G01750* | -5.08 | -2.49 | -6.57 | Actin depolymerizing factor 11 |
| *AT3G54770* | -5.15 | -4.44 | -6.33 | RNA-binding protein |
| *AT4G25790* | -5.05 | -3.12 | -6.65 | CAP superfamily protein |
| *AT5G05500* | -11.90 | -7.26 | -17.45 | Extensin family protein |
