# Supplementary material for: Salt-Induced Stabilization of EIN3/EIL1 Confers Salinity Tolerance by Deterring ROS Accumulation in Arabidopsis
Source: PLoS Genet. 2014 Oct 16;10(10):e1004664. doi: 10.1371/journal.pgen.1004664 (PMC4199496; doi:10.1371/journal.pgen.1004664)
Supplement: Table S3 — Genes up-regulated more than 5-fold in EIN3ox versus Col-0 under normal condition (62). Genes up-regulated more than 2-fold in salt-treated Col-0 were highlighted in red. (DOC) [file pgen.1004664.s018.doc]

**Table S3.** Genes Up-regulated More Than 5-fold in *EIN3ox* versus Col-0 Under Normal Condition (62). Genes up-regulated more than 2-fold in salt-treated Col-0 were highlighted in red.

| Locus | *EIN3ox*/Col | Col(+/-NaCl) | Description |
| --- | --- | --- | --- |
| **Transcription** | |  |  |
| *AT1G06160* | 6.08 | 6.16 | ORA59 |
| *AT1G28370* | 6.03 | 13.23 | ERF11 |
| *AT2G46990* | 9.34 | 1.03 | Auxin-induced protein (IAA20) |
| *AT3G20770* | 21.92 | 1.94 | Ethylene-insensitive 3 (EIN3) |
| *AT5G67060* | 6.99 | 1.86 | HECATE1 |
| **Oxidoreductase activity** | | |  |
| *AT1G49570* | 23.02 | -1.08 | Peroxidase superfamily protein |
| *AT1G69880* | 5.56 | -1.89 | Thioredoxin H-type 8 (TH8) |
| *AT1G80130* | 9.86 | -1.13 | Tetratricopeptide repeat (TPR)-like superfamily protein |
| *AT2G15560* | 63.35 | -1.02 | Putative endonuclease or glycosyl hydrolase |
| *AT5G13930* | 5.19 | -3.38 | Chalcone synthase |
| *AT5G19890* | 34.46 | -1.22 | Peroxidase ATP N |
| *AT5G44440* | 5.21 | -2.69 | Berberine bridge enzyme-like protein |
| **Defense** |  |  |  |
| *AT1G33790* | 11.17 | 2.13 | Myrosinase binding protein |
| *AT2G33130* | 6.01 | 3.76 | RALF-LIKE 18 |
| *AT4G16260* | 5.9 | -1.94 | Beta-1,3-glucanase class I precursor |
| *AT4G33720* | 50.77 | -1.13 | Pathogenesis-related protein 1 precursor |
| *AT5G09530* | 5.72 | -3.11 | Proline-rich protein 10 |
| **Biosynthetic process and metabolism** | | | |
| *AT1G01390* | 9.17 | 1.01 | Flavonol 3-o-glucosyltransferase |
| *AT1G23730* | 12.05 | -1.17 | Putative carbonic anhydrase |
| *AT1G47780* | 7.92 | -1.03 | Hypothetical protein |
| *AT1G50060* | 9.15 | -1.57 | Branched-chain amino acid aminotransferase |
| *AT1G50290* | 12.4 | -1.03 | Response to oxidative stress |
| *AT1G58025* | 19.13 | -1.01 | DNA-binding bromodomain-containing protein |
| *AT1G60810* | 5.47 | -2.55 | ATP citrate-lyase |
| *AT1G67810* | 7.23 | 12.5 | SUFE2 |
| *AT2G39980* | 8.99 | 6.76 | Putative anthocyanin 5-aromatic acyltransferase |
| *AT2G40100* | 8.8 | 1.19 | Putative chlorophyll a/b binding protein |
| *AT2G40960* | 16.83 | -1.12 | Single-stranded nucleic acid binding R3H protein |
| *AT3G21720* | 7.08 | -1.29 | Putative isocitrate lyase |
| *AT3G29410* | 7.31 | -1.07 | Terpene synthase |
| *AT4G11610* | 5.34 | -1.12 | Phosphoribosylanthranilate transferase |
| *AT4G35190* | 6.11 | 4.76 | Putative lysine decarboxylase family protein |
| *AT3G48580* | 9.15 | 3.39 | Endoxyloglucan transferase-like protein EXGT1 |
| *AT5G06570* | 19.34 | 4.67 | Alpha/beta-Hydrolases superfamily protein |
| *AT5G10770* | 5.69 | -2.04 | Nucleoid DNA-binding protein cnd41-like protein |
| *AT5G19100* | 7.69 | 2.09 | Dermal glycoprotein precursor -like protein |
| *AT5G24210* | 5.87 | 6.76 | Alpha/beta-Hydrolases superfamily protein |
| *AT5G49690* | 5.69 | 16.87 | UDP-Glycosyltransferase |
| **Transport** |  |  |  |
| *AT1G17810* | 11.54 | 2.78 | Tonoplast intrinsic protein |
| *AT1G61800* | 7.34 | 16.4 | Glucose-6-phosphate/phosphate-translocator precursor |
| *AT3G05150* | 12.29 | -3.88 | Putative sugar transporter similar to ERD6 |
| *AT3G24450* | 5.31 | -2.86 | Heavy metal transport/detoxification protein |
| *AT5G17700* | 6.56 | 1.54 | MATE efflux family protein |
| **Cell wall biogenesis** | |  |  |
| *AT3G44990* | 7.04 | -1.95 | Xyloglucan endo-transglycosylase |
| **Response to hormone** | |  |  |
| *AT1G52050* | 5.08 | -1.61 | Jasmonate inducible protein |
| *AT1G52060* | 10.5 | -2.16 | Jasmonate inducible protein |
| *AT1G52070* | 5.41 | -1.59 | Jasmonate inducible protein |
| *AT1G54040* | 128.12 | 2.56 | Epithiospecifier protein, interacts with WRKY53 |
| *AT4G21200* | 6.19 | 1.19 | Gibberellin 20-oxidase |
| **Regulation of cell proliferation** | | |  |
| *AT2G41230* | 9.01 | 2.96 | Organ size related 1(ORS1) |
| *AT3G59900* | 6.94 | -1.66 | Auxin-regulated gene involved in organ size |
| *AT5G54370* | 5.08 | -2.21 | Root cap protein 2-like protein |
| **Unknown** |  |  |  |
| *AT1G12030* | 20.39 | 1.19 | Hypothetical protein |
| *AT2G22510* | 5.18 | -2.35 | Unknown protein |
| *AT3G60070* | 7.07 | -1.48 | Putative protein |
| *AT3G60520* | 5.26 | 1.95 | Putative protein |
| *AT4G16442* | 5.07 | -1.58 | Expressed protein |
| *AT4G19240* | 9.03 | -1.06 | Hypothetical protein |
| *AT4G35720* | 8.51 | 4.33 | Putative protein |
| *AT5G15360* | 15.25 | -1.09 | Putative protein |
| *AT5G15420* | 12.58 | 1.01 | Putative protein |
| *AT5G26731* | 5.64 | -1.38 | Expressed protein |
