# Supplementary material for: Salt-Induced Stabilization of EIN3/EIL1 Confers Salinity Tolerance by Deterring ROS Accumulation in Arabidopsis
Source: PLoS Genet. 2014 Oct 16;10(10):e1004664. doi: 10.1371/journal.pgen.1004664 (PMC4199496; doi:10.1371/journal.pgen.1004664)
Supplement: Table S4 — Summary of genes, Salk T-DNA lines, insertion site, description and phenotype on salt medium. Numbers in the brackets are the exact positions of T-DNA insertion, and minus indicates that the insertion site is located in the upstream of coding region. Mutants exhibiting hypersensitivity to salt stress were highlighted in red. (DOC) [file pgen.1004664.s019.doc]

**Table S4.** Summary **of Genes**, Salk T-DNA Lines, Insertion Site, Description and Phenotype on Salt Medium. Numbers in the brackets are the exact positions of T-DNA insertion, and minus indicates that the insertion site is located in the upstream of coding region. Mutants exhibiting hypersensitivity to salt stress were highlighted in red. (DOC)

| Locus | Mutant | Insertion site | Description | Phenotype |
| --- | --- | --- | --- | --- |
| *AT1G05575* | SALK_069153 | Promoter (-135) | Expressed protein | None |
| *AT1G05680* | SALK_091130 | Promoter (-149) | Indole-3-acetate glucosyltransferase | None |
| *AT1G06620* | SALK_095793 | Intron 1 (36) | Oxidoreductase | None |
| *AT1G07400* | SALK_059920 | Promoter (-265) | Heat shock protein HSP17.8 | None |
| *AT1G15580* | SALK_112553 | Exon 1 (224) | IAA5 | None |
| *AT1G19180* | SALK_011957 | Exon 2 (195) | JAZ1 | None |
| *AT1G21110* | SALK_024048 | Exon 3 (88) | O-methyltransferase | None |
| *AT1G21120* | SALK_116939 | Exon 3 (262) | O-methyltransferase, | None |
| *AT1G23710* | SALK_041412 | Promoter (-392) | Unknown protein | None |
| *AT1G25400* | SALK_093560 | Exon 1 (680) | Unknown protein | None |
| *AT1G30700* | SALK_117810 | Exon 1 (101) | Putative reticuline oxidase | None |
| *AT1G35140* | SALK_132499 | Exon 1 (44) | Phosphate-induced (phi-1) | Sensitive |
| *AT1G54050* | SALK_072866 | Promoter (-482) | Heat-shock protein | None |
| *AT1G59860* | SALK_152961 | Exon 1 (19) | 17.6 kDa class I heat shock protein | None |
| *AT1G65510* | CS852423 | Exon 1 (73) | Hypothetical protein | None |
| *AT1G66570* | CS810227 | Promoter (-124) | Sucrose-proton symporter | None |
| *AT1G72060* | SALK_020586 | Exon 1 (18) | Unknown protein | None |
| *AT1G74950* | SALK_025279 | Exon 5 (7) | Unknown protein | None |
| *AT1G76600* | SALK_092167 | Promoter (-396) | Unknown protein | None |
| *AT2G18680* | SALK_001089 | Exon 1 (50) | Unknown protein | None |
| *AT2G25735* | SALK_052558 | Promoter (-586) | Expressed protein | None |
| *AT2G26530* | SALK_031880 | Exon 1 (678) | AR781, similar to yeast pheromone | None |
| *AT2G34600* | SALK_040835 | Promoter (-442) | JAZ7 | None |
| *AT2G38870* | SALK_111051 | Exon 1 (10) | Putative protease inhibitor | None |
| *AT2G40140* | SALK_024800 | Exon 1 (628) | SZF2 | Sensitive |
| *AT2G41100* | SALK_056345 | Promoter (-119) | Calmodulin-like protein | None |
| *AT2G43620* | SALK_056680 | Intron 1 (19) | Putative endochitinase | None |
| *AT3G19580* | SALK_132562 | Exon 1 (766) | AZF2 | Sensitive |
| *AT3G25655* | CS842296 | Exon 1 (156) | IDL1 | None |
| *AT3G51450* | SALK_124439 | Exon 4 (258) | Mucin –like protein hemomucin | None |
| *AT3G55970* | SALK_024417 | Intron 1 (52) | JA-REGULATED GENE 21 | None |
| *AT4G21830* | SALK_014786 | Promoter (-342) | ATMSRB7 | None |
| *AT4G21840* | SALK_019933 | Promoter (-409) | ATMSRB8 | None |
| *AT4G28460* | SALK_119896 | Promoter (-384) | Hypothetical protein | None |
| *AT4G37370* | SALK_129086 | Promoter (-531) | p450, CYP81D8 | None |
| *AT5G05600* | SALK_073705 | Promoter (-436) | Leucoanthocyanidin dioxygenase | None |
| *AT5G19110* | SALK_108042 | Exon 2 (200) | Dermal glycoprotein | None |
| *AT5G20230* | SALK_057903 | Exon 2 (609) | Senescence-associated gene 14 | None |
| *AT5G22270* | SALK_067396 | Intron 1 (63) | Putative protein | Sensitive |
| *AT5G26340* | SALK_021204 | Intron 2 (544) | SUGAR TRANSPORT 13 | None |
| *AT5G27420* | SALK_002809 | Promoter (-169) | ATL6/CNI1 | Sensitive |
| *AT5G40260* | SALK_092239 | Exon 5 (40) | SWEET8 | None |
| *AT5G40590* | SALK_104782 | Exon 1 (650) | Putative protein | None |
| *AT5G48430* | SALK_133276 | Exon 1 (1428) | Dermal glycoprotein precursor | None |
| *AT5G59820* | SALK_129023 | Promoter (-608) | ZAT12 | Sensitive |
| *AT5G64900* | SALK_089928 | Promoter (-199) | PRECURSOR OF PEPTIDE 1 | None |
| *AT5G67080* | CS873873 | Promoter (-396) | MAPKKK19 | None |
