# Supplementary material for: Salt-Induced Stabilization of EIN3/EIL1 Confers Salinity Tolerance by Deterring ROS Accumulation in Arabidopsis
Source: PLoS Genet. 2014 Oct 16;10(10):e1004664. doi: 10.1371/journal.pgen.1004664 (PMC4199496; doi:10.1371/journal.pgen.1004664)
Supplement: Table S5 — Primers used in this work. (DOC) [file pgen.1004664.s020.doc]

**Table S5.** Primers Used in This Work.

| **Primer Name** | **Primer Sequences (5'-3')** |
| --- | --- |
| **For qRT-PCR** | |
| *At3g20770* F | TGAGATGGGAATGTGTGGAAAC |
| *At3g20770* R | GAGCTCTAGACATTTTCTTCCT |
| *At1g02920* F | CTCAAAGATGGTGAACACAAGA |
| *At1g02920* R | GTGATTGCTCTTGATTCTGCCA |
| *At5g27420* F | CCGTGTCGGCGGGTCAACCCGG |
| *At5g27420* R | CTTCTGTGTCTTCACTTCTGAG |
| *At2g41730* F | GTCGTCACCAAGGCATCGTAAG |
| *At2g41730* R | GCAGAGGACGAGTCCGACCCGA |
| *At4g37370* F | CGTCACCACTTCTCGCCACGTC |
| *At4g37370* R | GGCAGTGTTGATTGCGGCGGTG |
| *At4G16260* F | CGCTGAGTTCGTACTCGTAA |
| *At4G16260* R | ATGTTGTGCTCCCTGCCAT |
| *At5G54370* F | AAAGTGTGCTACGCCGATTG |
| *At5G54370* R | CCATTGATCTGAAGGTCAG |
| *At5G19890* F | TCTTCCTAATGCCACTCTCC |
| *At5G19890* R | ATGGAGCTGACAGCGAGAAA |
| *At4G33720* F | TAGCAGCAGTTGACATGTGG |
| *At4G33720* R | GAGGATCGTAGTTGCAAGTG |
| *At1G06160* F | AGAGACTCAACGAGGAAAGG |
| *At1G06160* R | TATCACCGGAGACTCTCCAT |
| *At1G28370* F | ACCGACGAAGAATCCGATTC |
| *At1G28370* R | CTTGAGTTTGACACAGAGCC |
| *At1G54040* F | TTGCAAGGCCAGTGGATCAA |
| *At1G54040* R | TCCTTTGGGTTGAGCGATTG |
| *At1G49570* F | CGATTCTATGACCGGTCTTG |
| *At1G49570* R | TTTGGCTGAGCGTTCTTCTC |
| *At3G20770* F | GGTAATAACCCGATTGGACC |
| *At3G20770* R | AAACCAAGTTGAGGCCACCA |
| *At1G17810* F | GGGTTGCTCAGCTTATAGGT |
| *At1G17810* R | GCGGTCGAGTAGACAACATA |
| *At1G52050* F | TTTCAGCCGACGGTTTCA |
| *At1G52050* R | CCATCGACTCCAAGTGAA |
| *At2G46990* F | TACCGCGACTTGATCAGAAC |
| *At2G46990* R | GACAAGAACATCTCCCAAGG |
| *At2G39980* F | CACACCTCAAACACTCTC |
| *At2G39980* R | CTCTTTGACTACATCGGG |
| *At2G40100* F | GAGGCTCTTACAGGAATTGC |
| *At2G40100* R | TCAAAATACCCACCCGGGTA |
| *At5G49690* F | GTCAGTAGGAGGGTTCTTGA |
| *At5G49690* R | AGCGACCGAGTCAGAATCAA |
| *At1G60810* F | GCTCGGAAGAAGATCAGAGA |
| *At1G60810* R | TTGCCACGCTTTCCGAACAA |
| *At5G44440* F | TACCCGAGGCTGCAATTCAA |
| *At5G44440* R | CTTGTCTTCCTCTTCTCTCC |
| *β-ACTIN2* F | TGCTGAGCTTATCGATTCCG |
| *β-ACTIN2* R | TTCGGTGATGGGAATACAG |
| **For PCR genotyping** | |
| Lb1.3 | ATTTTGCCGATTTCGGAAC |
| CS852423LP | CACCAATCGTGAACGTATGTG |
| CS852423RP | TTCAAATCAGCCAAAGATTCC |
| CS810227LP | CACTTGTTAGTGCTACAAAATTTTAGTG |
| CS810227RP | AATGAAAGATGGCCATTTGTG |
| CS842296LP | CCATTACTTGCGCTTCACTTC |
| CS842296RP | CAGAAGGTTTGATGAGAAGCG |
| CS873873LP | ACACCTTCACCGGCTAATTTC |
| CS873873RP | AGAGAGTCCAGAGCATAGGCC |
| SALK_069153LP | CATGGCAGAAGGAGACATCTC |
| SALK_069153RP | GCATGGAGTGGCATAAAGATG |
| SALK_091130LP | CGTTTTTCCGTTAAATATTTTAAATG |
| SALK_091130RP | AAGACCTTTTGAGGCTAAGCG |
| SALK_095793LP | TGATGGGTCCATAAACTCGAG |
| SALK_095793RP | CTCCTCTGATTTCTCCATCCC |
| SALK_059920LP | TGAATTTGTCAAGTAACCGCC |
| SALK_059920RP | TTGCAGTTCCTTGAATGGATC |
| SALK_112553LP | AAGGCTCACTCACATTCACATG |
| SALK_112553RP | CGGCTGAGAAAGAAACCCTAC |
| SALK_011957LP | AGGTAAATGCGGAGAGAGAGG |
| SALK_011957RP | AGGCACCGCTAATAGCTTAGC |
| SALK_024048LP | AGGAGCAGAGGGTCTATCGAG |
| SALK_024048RP | GAAAGCAGCTGGTCTTGTGAC |
| SALK_116939LP | CCTCCCTGCTTAAAATTTTGG |
| SALK_116939RP | ACCGACGAAGACTGTGTGAAG |
| SALK_041412LP | GTTCTTTCTCAGAACCCTCCG |
| SALK_041412RP | AAACATATGGACCAAAATCGTTG |
| SALK_093560LP | TCCAGCTAATTGTCTTTTCCG |
| SALK_093560RP | GTAATTACGACAGCGACTCCG |
| SALK_117810LP | ATATCCTCCCCATCTCACCAC |
| SALK_117810RP | GAAACCCTTCCTCATAATCGC |
| SALK_072866LP | GTTCAGAGAATCGATCTCCCC |
| SALK_072866RP | GGGGTTTGAAATTTTCACTCC |
| SALK_152961LP | TTGGTGGCCACTTAGAAGAAG |
| SALK_152961RP | CCGGTAACCTAAACTTCCTCG |
| SALK_020586LP | TCACCAAAGTCCCACAAAAAG |
| SALK_020586RP | AACCCAGGAGAAGCAGAAATC |
| SALK_025279LP | AGCCTGGTCTGATCTACTCCAC |
| SALK_025279RP | TCTACGGTGGTCGAGTTATGG |
| SALK_092167LP | GCAAACAGCTTTCTCATCCTG |
| SALK_092167RP | TTGTGGATCGAGTTCAGCTTC |
| SALK_001089LP | TCGTTGGAGGAGACTAATTGC |
| SALK_001089RP | TCGTTGGAGGAGACTAATTGC |
| SALK_052558 LP | CATTGAAAGGTTGGCGTAGTG |
| SALK_052558 RP | CAGTCGTCGTCGATTGATTTC |
| SALK_031880LP | GAAGTGATGAGCTTGACTGCC |
| SALK_031880RP | GTTGGGATCGAAGATAGAGGG |
| SALK_040835LP | CAAACAAACTTCACATCAATCCC |
| SALK_040835RP | CGAGACAAAAGCTGGCATTAG |
| SALK_111051LP | TTGTCTACACGTTGCCACTTG |
| SALK_111051RP | TGGTCATTTTTGTCGGCTAAG |
| SALK_056345LP | TCAGATTCTTTACATTGCGGC |
| SALK_056345RP | CTGAAGATCAGCTTTTGTCGG |
| SALK_056680LP | ATGACCGACATGGAATTTCTC |
| SALK_056680RP | AGTCAAGAGAGGGAGTCCGAG |
| SALK_124439LP | TGCCAGTAAACGGAATCAAAC |
| SALK_124439RP | GAGCTCACCAGTTAGCGACAC |
| SALK_024417LP | ACCTATGCATGCCCTCTGTAG |
| SALK_024417RP | AAACAGGGAGTCACGACATTG |
| SALK_014786LP | CAACCGGAGTCGAACTTAGTG |
| SALK_014786RP | TCGCATAGTTTCACCATTTCC |
| SALK_019933LP | ATCCACTTGATGGAACTGCAG |
| SALK_019933RP | TGTGACTGAGAGATTTTCAAGGG |
| SALK_119896LP | TAATTAATCTCCTCCGGCCAG |
| SALK_119896RP | AGTCTTATTTTGGTGGGGACG |
| SALK_129086LP | CCGAGAAAGACGAAACACAAG |
| SALK_129086RP | TTTAGCAACAAACTCACACAAGC |
| SALK_073705LP | TGTTTGATGTGCAACAATAACAG |
| SALK_073705RP | AAGGGTAAAATGGTGGTGGAG |
| SALK_108042LP | TCCCACCATTCAATAGTAGCG |
| SALK_108042RP | ATTAATGTGCAAAATCCTCAGAC |
| SALK_057903LP | AGTTTCCACCGACTGATGTTG |
| SALK_057903RP | TGATTCACGTCGATCAGACAC |
| SALK_021204LP | TTATTGCAGGCAGGATATTGC |
| SALK_021204RP | CTGAGGACGGTTTCTACGTTG |
| SALK_092239LP | GGAAGACTTTCTGGAGGATCTTC |
| SALK_092239RP | CGAATCCTCAAAATCCAGAAAC |
| SALK_104782LP | AAGCTCGAATCGGAGAAGAAG |
| SALK_104782RP | AGTTTCCAAATGCGATGTTTG |
| SALK_133276LP | TTGGGACTCAAGGGTATTTCC |
| SALK_133276RP | GGTTTTTCGAAAATATCTTGCC |
| SALK_089928LP | CTTCAACAACATTGTGATAGGC |
| SALK_089928RP | TAGGTGACTTTCTTCGCTTCG |
| SALK_132499LP | GGTTCTCCCTGTTTCAACTCC |
| SALK_132499RP | GATCTGTTCTCCGAGGGAGAG |
| SALK_024800LP | CAATTTGCGTTGCTCTTTCTC |
| SALK_024800RP | TCTGTTCTGCCATAAACCACC |
| SALK_132562LP | TTGTTGCTAACAAGCATGTGC |
| SALK_132562RP | GCCAGAATCAAAGAACCTTCC |
| SALK_067396LP | TGAGGACACAATGACACATCC |
| SALK_067396RP | TGCAATTTCAAAGCACAAGTC |
| SALK_002809LP | AATTTGGTTTGCTTTTGGGAC |
| SALK_002809RP | GTAACGCTACCGTCTACTGCG |
| SALK_129023LP | TGGAACGAATGAAACTGCTTC |
| SALK_129023RP | CCTTTGCTGGGATTGTGTTAG |
